# Structural water and disordered structure promote aqueous sodium-ion energy storage in sodium-birnessite

**DOI:** 10.1038/s41467-019-12939-3

**Published:** 2019-10-31

**Authors:** Xiaoqiang Shan, Fenghua Guo, Daniel S. Charles, Zachary Lebens-Higgins, Sara Abdel Razek, Jinpeng Wu, Wenqian Xu, Wanli Yang, Katharine L. Page, Joerg C. Neuefeind, Mikhail Feygenson, Louis F. J. Piper, Xiaowei Teng

**Affiliations:** 1 Department of Chemical Engineering, University of New Hampshire, Durham, NH 03824 USA; 2 Department of Physics, Applied Physics and Astronomy, Binghamton University, Binghamton, NY 13902 USA; 3 Advanced Light Source, Lawrence Berkeley National Laboratory, Berkeley, CA 94720 USA; 4 Advanced Photon Source, Argonne National Laboratory, Argonne, IL 60439 USA; 5 Chemical and Engineering Materials Division, Spallation Neutron Source, Oak Ridge National Laboratory, Oak Ridge, TN 37831 USA; 6 Juelich Centre for Neutron Science, Forschungszentrum Juelich GmbH, 52425 Juelich, Germany

**Keywords:** Energy science and technology, Materials science

## Abstract

Birnessite is a low-cost and environmentally friendly layered material for aqueous electrochemical energy storage; however, its storage capacity is poor due to its narrow potential window in aqueous electrolyte and low redox activity. Herein we report a sodium rich disordered birnessite (Na_0.27_MnO_2_) for aqueous sodium-ion electrochemical storage with a much-enhanced capacity and cycling life (83 mAh g^−1^ after 5000 cycles in full-cell). Neutron total scattering and in situ X-ray diffraction measurements show that both structural water and the Na-rich disordered structure contribute to the improved electrochemical performance of current cathode material. Particularly, the co-deintercalation of the hydrated water and sodium-ion during the high potential charging process results in the shrinkage of interlayer distance and thus stabilizes the layered structure. Our results provide a genuine insight into how structural disordering and structural water improve sodium-ion storage in a layered electrode and open up an exciting direction for improving aqueous batteries.

## Abstract (web summary)

The authors report a sodium rich disordered birnessite cathode material Na_0.27_MnO_2_ for aqueous Na-ion storage. The impressive electrochemical performance is attributed to the co-deintercalation of structural water and Na-ion during the high potential charging process which stabilizes the layered structure.

## Introduction

Electrochemical energy storage (EES) using earth-abundant materials has become attractive for storing electric energy generated by solar and wind^1^. Aqueous EES using sodium (Na)-ion as charge carrier is promising alternative to non-aqueous lithium (Li)-ion batteries (LIBs) owing to low cost, high safety and the availability of Na sources in terrestrial reserves^2^. However, Na-ion storage is challenging for its large radius. Consequently, LIB host materials (especially cathode) that typically have a close-packed array of oxide ions are able to reversibly accommodate Na-ions. Two design principles are used to tackle the issue. One is to replace oxygen anions (O^2−^) with anions having weaker bonding with metal cations so that cations are sufficiently mobile in the electrode. Recent studies show promise of hexacyano ion (C≡N)_6_^6‒^ based electrode materials for Na- and K-ion storage due to the weakened bonding between cyanide (C≡N)^‒^ and cations. Cui and co-workers have demonstrated that potassium copper hexacyanoferrate and its analogues can function as stable electrode materials for aqueous K- and Na-ion storage^3–5^. Sodium-manganese hexacyanoferrate reported by Goodenough’s group showed good energy performance and cycling life in a non-aqueous electrolyte^6–8^. Another approach is to use a large interstitial host framework, especially layered structure. Layered materials with planar or zigzag layers show different polymorphs (P2, P3 or O2, O3 phase) with respect to the sites of intercalated Na-ion by simply altering the stacking of transition-metal-oxygen octahedra^9–11^. Pioneer works on studying Na-ion intercalation in layered Na_x_MnO_2_ were reported by Hagenmuller in the 1980s^12,13^. However, the mechanistic understanding of Na-ion storage inside various host materials is still not settled. Size difference between Na-ion and Li-ion gives rise to different intercalation chemistries^14^, so that the understanding obtained from Li-ion storage may not be directly applied to the Na-ion electrodes^15–18^. In addition, layered Na_x_MnO_2_ especially P-type materials where Na-ions occupy trigonal prismatic site suffer from P2-O2 phase transition during charging with a large lattice collapse and up to 23% of volume shrinkage, resulting in an increased Na-ion diffusion barrier and rapid capacity decay^19,20^.

Birnessite (δ-MnO_2_) is a layered structure comprised of two-dimensional sheets of edge-sharing MnO_6_ octahedra with intercalated cations and/or water in the interlayer^21–25^. Although birnessite has a large interlayer distance (~7 Å), its storage capacities for Na-ion were low due to the limited thermodynamically stable potential window (~1.23 V) of an aqueous electrolyte and ineffective redox process^26–28^. Recent studies showed that a concentrated Li-bis(trifluoromethane sulfonyl)imide salt in water electrolyte helped the formation of an electrode-electrolyte interphase on a Mo_6_S_8_ anode that prevented the direct contact between anode and water, thus achieved potential window of 3.0 V for aqueous EES^29^. A similar wide potential window was reported for aqueous electrolyte using hydrate-melt of Li salts^30,31^. However, little work has been reported to date on how to widen the potential window of birnessite electrode materials in aqueous electrolytes.

Here we present an effective strategy to significantly improve the discharge capacity and cycle life of birnessite (full-cell capacity of 83 mAh g^−1^ at 1 A g^−1^ after 5000 cycles) through increasing the stable potential window and promoting redox charge transfer process towards aqueous Na-ion storage. Our results demonstrate that Na-rich and disordered birnessite structure can afford a stable potential window of 2.5 V in an aqueous electrolyte with high overpotential towards the gas evolution reactions. Moreover, co-deintercalation of water molecules along with Na-ion at the high potential charging, evidenced by in situ XRD, can stabilize the layered structure from over-expansion of the interlayer distance.

## Results

### Structural characterizations and formation mechanism

**Fig. 1:**
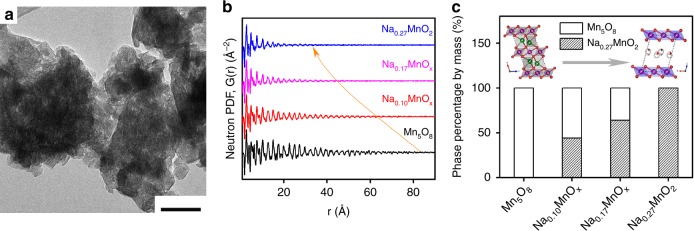
TEM and neutron PDF analysis of sodium-manganese oxides. **a** TEM image of of Na_0.27_MnO_2_ materials; scale bar, 50 nm; **b** Experimental neutron PDFs of Na_δ_MnO_x_ materials, where coherent lengths, defined as the longest interatomic distance of the material, decreased as the Na concentration increased; **c** Phase percentage of Na_0.27_MnO_2_ in Na_δ_MnO_x_ materials obtained from neutron PDF analysis

Different from wet chemistry synthesis of birnessite^24,25,27,32^, disordered and Na-rich birnessite were prepared at 270 °C in air via a solid-state reaction between NaOH and Mn_3_O_4_ nanoparticles (Mn_3_O_4_ particles were synthesized using a method reported previously^33^). By altering the molar ratios between NaOH and Mn_3_O_4_, sodium-intercalated manganese oxides (Na_δ_MnO_x_; δ: 0.10, 0.17, and 0.27) were prepared, verified by Inductively Coupled Plasma Mass Spectrometry (ICP-MS) (Supplementary Table 1). The Na/Mn ratio remained around 0.27 even when a higher NaOH/Mn_3_O_4_ ratio of 4 was used (Supplementary Figure 1). The Na/Mn ratio of 0.27 is higher than those of birnessite materials reported (Supplementary Table 2)^21–25^. Figure 1a and Supplementary Fig. 2 show that as Na concentrations increased from 0.10 to 0.27, the morphologies of resulting Na–Mn–O materials evolved from a mixture of faceted particles and planar structures to a complete planar structure. The atomic ordering of the resulting Na–Mn–O materials was analyzed using neutron total scattering and the atomic pair distribution function (PDF) (Fig. 1b), from which both the Bragg and diffuse scattering were analyzed to provide local structural details such as defects, mismatch or disorder at the atomic level. The structural parameters of various Na_δ_MnO_x_ materials obtained from refinement are summarized in Supplementary Fig. 3 and Supplementary Table 3–6. The neutron PDF showed that the coherent length of Na_δ_MnO_x_ materials (a distance at which the peaks of atomic pairs vanished) decreased from >50 Å to ~30 Å as δ increases, indicating more confined crystalline order. Namely, Na_δ_MnO_x_ materials cannot sustain long-range crystallinity and became more disordered when more Na-ions are incorporated into the structure. Figure 1c shows that as Na concentration (δ) increased, Na_δ_MnO_x_ materials showed pure phase Mn_5_O_8_ (δ = 0), mixture of Mn_5_O_8_ and layered MnO_2_ (δ = 0.10 and 0.17). When δ reached 0.27, a pure triclinic birnessite structure formed with an interplanar distance of 7.19 Å. The chemical formula was determined as Na_0.27_MnO_2_∙0.63H_2_O, where the Na^+^ and structural water (determined from thermal gravimetric analysis shown in Supplementary Fig. 5) occupied the interlayers.

**Fig. 2:**
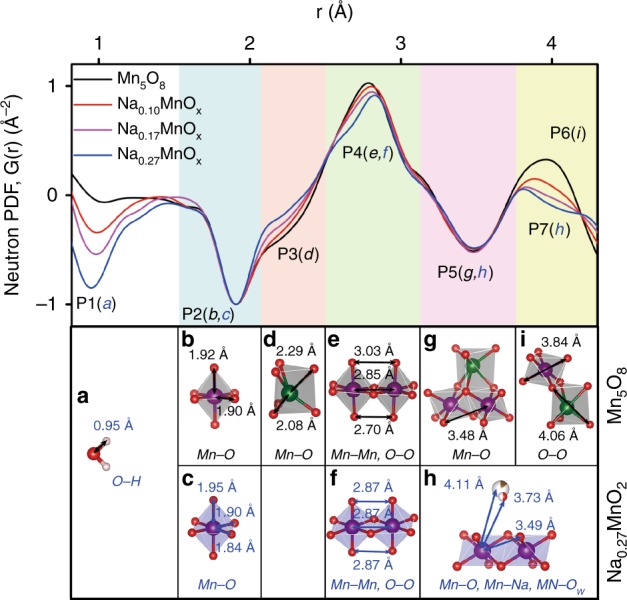
Neutron PDFs of various Na_δ_MnO_x_ materials. The atom pair associated with each peak (P1 to P7) can be attributed to **a** water, **b**, **d**, **e**, **g**, **i** Mn_5_O_8_ polyhedra in black and **c**, **f**, **h** MnO_2_ polyhedra in blue

Neutron PDF studied the evolution of local structure during the transition from Mn_3_O_4_ to Mn_5_O_8_, and finally to MnO_2_ birnessite (Fig. 2). The PDF data were normalized by the intensity of the peak at 1.9 Å to see the comparative changes of the structural details as δ increased, and the original coherent scattering and structure factor data can be found in Supplementary Fig. 6. The peaks of PDF can be indexed as O–H pair (0.95 Å, P1) from water (*a*), one Mn–O pair (1.9 Å, P2) from the [MnO_6_] octahedral unit and another Mn–O pair (2.2 Å, P3) from Mn atoms in prismatic sites, Mn–Mn or O–O pair (2.8 Å, P4), and Mn–O pair (3.5 Å, P5) from the nearest neighbors of [MnO_6_] octahedral units, respectively. The contribution of individual pairs such as Mn–O, Mn–Mn, O–O, and Mn–Na, to the overall PDF of the materials can be found in Supplementary Figure 7. Notably O–H pair (P1) and Mn–O pairs (P2, P3, P5, and P7) showed negative peaks due to negative coherent neutron scattering lengths of H and Mn atoms (−3.74 and −3.73 femtometer, respectively). The Mn–O pair (1.9 Å, P2) is attributed to Mn-O octahedral coordination in both Mn_5_O_8_ (*b*) and layered Na_0.27_MnO_2_ (*c*) structures. The Mn–O pair (2.2 Å, P3) is attributed to Mn(II)–O from Mn_5_O_8_ phase (*d*), which decreased relatively to Mn(IV)–O pair at P2 as δ increased, congruent with the decreasing phase fractions of Mn_5_O_8_. The positive peaks at 2.8 Å (P4) are attributed to Mn–Mn or O–O bonding from adjacent [MnO_6_] octahedral units in Mn_5_O_8_ (*e*) and Na_0.27_MnO_2_ (*f*) relative to the Mn(IV)–O pair, which did not change significantly as δ increased. A similar trend can be found in the Mn–O pair at 3.5 Å (P5) from adjacent [MnO_6_] in Mn_5_O_8_ (*g*) and Na_0.27_MnO_2_ phases (*h*). The peaks at ~4.0 Å (P6 and P7) showed a rather interesting transition from positive to negative direction as δ increased. The positive peak at 3.96 Å (P6) is related to O–O pairs (*i*) in Mn_5_O_8_ either within the same [Mn(IV)O_6_] octahedral unit or [Mn(II)–O] units where Mn^2+^ is located in the trigonal prismatic site. In contrast, the negative peak at 4.0 Å (P7) is attributed to Mn–Na pair at 4.11 Å (*h*) from the interaction between Na-ions in interlayers and Mn^4+^ from [MnO_6_] octahedra or Mn–O_w_ pair (O_w_ from structural water in the interlayer) at 3.73 Å (*h*) from the interaction between H_2_O in interlayers and Mn^4+^, both from Na_0.27_MnO_2_ layered phase. The interplay between negative peaks of Mn–Na and Mn–O_W_ in Na_0.27_MnO_2_ and the positive peak of O–O in Mn_5_O_8_ at around 4.0 Å explains the overall peak change from positive to negative directions when δ increased. This suggests that phase transition from Mn_5_O_8_ to Na_0.27_MnO_2_ birnessite was driven by Na-ion insertion during solid-state annealing.

**Fig. 3:**
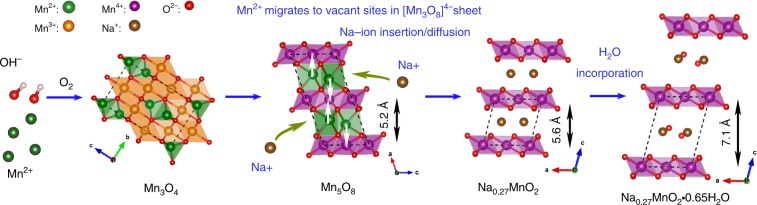
The schematic of the formation mechanism. The proposed Na-ion driven intercalation in the formation of Na_0.27_MnO_2_·0.65H_2_O materials in solid-state (Mn^2+^: green; Mn^3+^: orange; Mn^4+^: purple; Na^+^: brown; O: red)

From neutron PDF and in situ XRD during the thermal treatment (Supplementary Fig. 8), a formation mechanism of Na_0.27_MnO_2_ birnessite is proposed in Fig. 3. Mn_3_O_4_ nanoparticles were first converted into Mn_5_O_8_ materials through oxidation of [Mn(III)O_6_] octahedra of Mn_3_O_4_ into [Mn (IV)O_6_] units, followed by Na-ion driven conversion from Mn_5_O_8_ to Na_0.27_MnO_2_ birnessite during the thermal annealing in air. Mn_5_O_8_ and Na_0.27_MnO_2_ share similar structural characteristics: Mn_5_O_8_ has a layered structure and consists of sheets of $[{\mathrm{Mn}}_3^{4 + }{\mathrm{O}}_8]^{4 - }$ in the *bc* plane. The $[{\mathrm{Mn}}_3^{4 + }{\mathrm{O}}_8]^{4 - }$ sheets resemble the structure of Na_0.27_MnO_2_ birnessite comprised of infinite [MnO_6_] octahedral layers with intercalated Na cations in between. The transition from Mn_5_O_8_ to Na_0.27_MnO_2_ birnessite is an equivalent process to the ion-exchange of Mn^2+^ ions in the ${\mathrm{Mn}}_2^{2 + }{\mathrm{Mn}}_3^{4 + }{\mathrm{O}}_8$ with Na^+^ ions in the solid state. Our result suggests that Mn^2+^ ions with trigonal prismatic coordination located in interlayers of Mn_5_O_8_ have a higher mobility than octahedrally coordinated Mn^4+^ ions. Therefore, insertion of Na-ions into the Mn^2+^ site was kinetically favored, accompanied by the oxidation of Mn^2+^ ions into Mn^4+^ during the migration of Mn^2+^ to the [Mn^4+^_3_O_8_]^4−^ layers, and drove the formation of Na_0.27_MnO_2_. XRD showed that anhydrous Na_0.27_MnO_2_ has an interlayer distance of 5.6 Å (Supplementary Fig. 9), similar to that of Mn_5_O_8_ (5.2 Å). Upon water intercalation, Na_0.27_MnO_2_·0.63H_2_O showed an increased interlayer distance of 7.19 Å^34^. Na-ion driven conversion from Mn_5_O_8_ to Na_0.27_MnO_2_ reported here contrasts the formation of Li-MnO_2_ via the ion-exchange between Ca_2_Mn_3_O_8_ (Ca^2+^_2_Mn^4+^_3_O_8_), isomorphic structure of Mn_5_O_8_ (Mn^2+^_2_Mn^4+^_3_O_8_), and molten lithium nitrate^35^. In the formation of Li-MnO_2_, Li-ions occupied all the available octahedral sites between the $[{\mathrm{Mn}}_3^{4 + }{\mathrm{O}}_8]^{4 - }$ layers rather than the trigonal prismatic sites occupied by Ca^2+^ in the parent Ca_2_Mn_3_O_8_ compound due to much smaller size of Li^+^ compared with Ca^2+^, resulting in the complete conversion to layered LiMnO_2_ with ${\mathrm{R}}\bar 3{\mathrm{m}}$ or O3 symmetry.

### Electrochemical properties

**Fig. 4:**
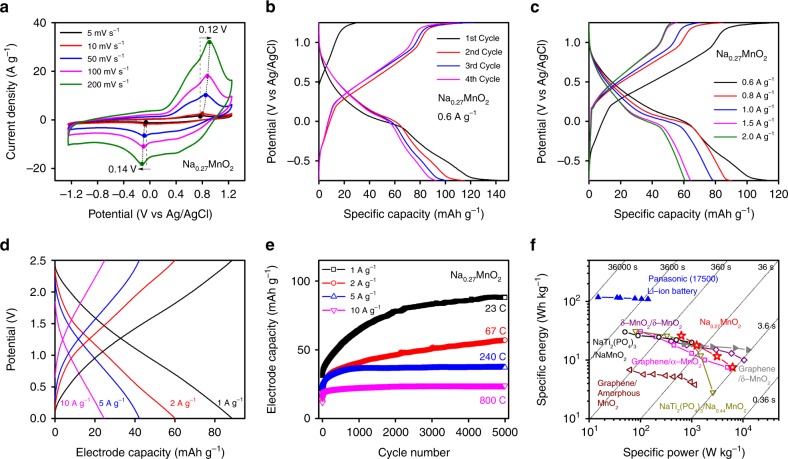
Electrochemical measurements of Na_0.27_MnO_2_ in half-cells and full-cells. Electrochemical half-cell measurements with **a** CV scans of Na_0.27_MnO_2_ material between −1.25 V and 1.25 V (vs Ag/AgCl) at various scan rates in 0.1 M Na_2_SO_4_ electrolyte; **b** CP tests of Na_0.27_MnO_2_ material between −0.75 V and 1.25 V (vs Ag/AgCl) at a current density of 0.6 A g^−1^ in 0.1 M Na_2_SO_4_ electrolyte with the intial four charge and discharge cycles **c** CP tests of Na_0.27_MnO_2_ material between −0.75 V to 1.25 V (vs Ag/AgCl) at current densities ranging from 0.6 to 2.0 A g^−1^ in 0.1 M Na_2_SO_4_ electrolyte (2nd cycle data). Symmetric full-cell measurements with **d** charge and discharge electrode capacities of Na_0.27_MnO_2_ material at the various current densities of 1 A g^−1^, 2 A g^−1^, 5 A g^−1^, and 10 A g^−1^ (after 5000 galvonstatic charge and discharge process unless specified otherwise); **e** electrode capacities of Na_0.27_MnO_2_ as a function of cycle number up to 5000 at the current densities from 1 A g^−1^ to 10 A g^−1^; **f** Ragone plot with gravimetric specific energy and power of the symmetric Na_0.27_MnO_2_ full-cell after 5000 galvanostatic cycles. The aqueous (empty symbols) and non-aqueous (solid symbols) devices are reported, and the gravimetric specific energy and power are calculated by the mass of electrode materials except the Panasonic (17500) Li-ion batteries

Electrochemical performance of various Na_δ_MnO_x_ materials was tested in a 0.1 M Na_2_SO_4_ electrolyte in a three-electrode half-cell using cyclic voltammetry (CV) between −1.25 V and 1.25 V (vs Ag/AgCl) (Supplementary Fig. 10). Figure 4a shows the CVs of disordered Na-rich Na_0.27_MnO_2_, where distinct redox peaks can be observed at all the tested scan rates. Figure 4a shows that when scan rate increased, Na_0.27_MnO_2_ shows small peak shifts in the anodic process (0.12 V) and the cathodic process (0.14 V), compared with the other Na_*δ*_MnO_*x*_ materials (Supplementary Fig. 11), indicating Na-ion transport in Na_0.27_MnO_2_ required a lower overpotential at higher charging rates. Further quantitative evaluation of Na-ion transport in all Na_*δ*_MnO_*x*_ materials was obtained using a current-pulse relaxation method^13^, where the Na_0.27_MnO_2_ material showed a highest diffusion coefficient of 38.7 (relative to Mn_5_O_8_) than the other Na_δ_MnO_x_ materials (Supplementary Figure 12). This result was congruent with CV data, where Na_0.27_MnO_2_ had the lowest energy barrier for Na-ion intercalation since it had a more dominant phase of layered birnessite.

Figure 4b shows the galvanostatic chronopotentiometry (CP) tests of Na_0.27_MnO_2_ materials in half-cell at 0.6 A g^−1^ under the potential window between −0.75 V and 1.25 V (vs Ag/AgCl), demonstrating a high discharge capacity of 138 mAh g^−1^ (1st cycle) and a capacity of 94 mAh g^−1^ after 4th cycle. A highest discharge capacity of 144 mAh g^−1^ was observed for Na-ion storage at a current density of 0.3 A g^−1^ (Supplementary Fig. 13), although additional capacity could be attributed to the hydrogen evolution reaction (HER). It is suggested that discharge capacity of 144 mAh g^−1^ could be the highest capacity of Na_0.27_MnO_2_ materials measured at three-electrode half-cell conditions, corresponding to 0.47 Na-ion transfer per Mn atom. As Na_0.27_MnO_2_ electrode materials was initially charged to 1.25 V from open circuit voltage of ~0.5 V with the 1st cycle charge capacity of 71 mAh g^−1^, indicating a roughly 0.23 Na-ion removal. In the following 1st cycle discharge process, the MnO_2_ birnessite electrode displayed a discharge capacity about 144 mAh g^−1^, pointing to 0.47 Na-ion insertion. Figure 4c shows CPs of Na_0.27_MnO_2_ in the three-electrode half-cell at current densities from 0.6 A g^−1^ to 2 A g^−1^. As current densities increased, discharged capacities decreased from 115 to 61 mAh g^−1^. Moreover, Supplementary Figure 14 shows that the charge and discharge potential differences at the midpoint of the capacity increased from 0.65 V to 0.75 V as current densities increased, suggesting an increasing polarization for Na-ion transport, comparable to the values reported in Zn-MnO_2_^36–38^, Na–MnO_2_^39^, and Li_2_Mn_2/3_Nb_1/3_O_2_F system^40^, reflecting the intrinsic redox barrier for the MnO_2_ based electrode systems.

Supplementary Figure 15 shows X-ray photoelectron spectroscopy (XPS) spectra of Na_0.27_MnO_2_. The pristine state Mn showed a dominant Mn^4+^ state with characteristic Mn 2p_1/2_ and Mn 2p_3/2_ features at 654.2 eV and 642.5 eV, and less distinct but discernable Mn^3+^ features at 642.2 eV and 653.3 eV, respectively (Supplementary Fig. 16). The ratio between Mn^4+^ and Mn^3+^ was calculated to be 0.72:0.28. Since the Mn^3+^ resulted from the intercalated Na-ion in interlayers of MnO_2_, a Mn^4+^/Mn^3+^ ratio of 0.72:0.28 suggested a Na/Mn ratio of 0.28, nearly identical to the ICP-MS result (Na:Mn = 0.27). At charged state (1.25 V) Na_0.27_MnO_2_ showed a Mn^4+^/Mn^3+^ ratio of 0.97:0.03, and a Mn^4+^/Mn^3+^ ratio of 0.62:0.38 at discharged state (−1.25 V). Although the exact determination of Mn^2+/3+/4+^ ratio is challenging from the Mn 2p core region due to the multiplet structure and significant overlap between different oxidation states, XPS results confirmed that Na_0.27_MnO_2_ materials had a Mn^4+^/Mn^3+^ redox couple during insertion and extraction of Na-ions.

We also analyzed the current (*i*) at different scan rates (*v*) at a given potential, assuming that the total current (*i*) at a particular potential contains both capacitive current (*i*_1_ = *k*_1_*v*) and diffusion-limited redox current (*i*_2_ = *k*_2_*v*^1/2^):^41^

$$i = i_1 + i_2 = k_1v + k_2v^{1/2}\;{\mathrm{or}}\;i/v^{1/2} = k_1v^{1/2} + k_2$$

The values of *k*_1_ and *k*_2_ and] the relative current response from *i*_1_ and *i*_2_ can be obtained. The CVs marked with capacitive and diffusion-limited redox contributions at scan rates ranging from 5 to 1000 mV s^−1^ can be found and summarized in Supplementary Figs. 17 and 18.

Long-term performance of Na_0.27_MnO_2_ was tested in symmetric full-cells in a potential window of 2.5 V. Toray paper was used as the current collector without causing gas evolution reactions (Supplementary Fig. 19). Figure 4d and Supplementary Fig. 20 show that voltage-capacity profiles are nearly linear at all the tested current densities, pointing to a single-phase solid solution reaction. Accordingly, electrode capacities were calculated to be 83 mAh g^−1^ to 24 mAh g^−1^ at corresponding discharge times ranging from 160 s to 4.5 s. Moreover, Na_0.27_MnO_2_ exhibits an excellent cycle stability up to 5000 cycles without obvious capacity loss, as well as nearly 100% coulombic efficiency and high energy efficiency at various current densities (Fig. 4e). Figure 4f compares the energy and power performance of Na_0.27_MnO_2_ materials with several aqueous or non-aqueous EES devices, including Panasonic (17500) Li-ion battery^42^, α-MnO_2_, δ-MnO_2_ or amorphous birnessites^32,43–45^, and tunnel-structured Na_0.44_MnO_2_ and O3 type Na_x_MnO_2_^39,46^_._ Notably, energy and power densities of our reported system were obtained after 5000 galvanostatic cycles, higher than those found in commercial products and recent literature. Comparisons between current works with other Mn-based electrode materials in aqueous storage are summarized in Supplementary Table 7 ^43,46–48^.

Proton (H^+^) insertion has been reported in aqueous EES in mild acidic aqueous electrolytes^36,37^. Current work used a neutral Na_2_SO_4_ electrolyte, thus the H^+^ concentration was very low and H^+^ intercalation may not contribute to overall storage capacity. In addition, Na_0.27_MnO_2_ materials showed continuously increasing capacities during the initial cycling especially at the low current densities. The electrochemical impedance spectroscopy measurements (Supplementary Fig. 21) demonstrate decreasing solution resistance during the initial electrochemical cycling, congruent with I-R drop in the discharge curves (Supplementary Fig. 22). It suggested that the increasing capacities in early cycles can be attributed to the slow building-up of a transport before the electrode reached its best electrochemical condition. Similar behaviours were also observed in Na-S, Na_0.67_Ni_1/6_Co_1/6_Mn_2/3_O_2_, and LiFe_0.9_P_0.95_O_4_^49–51^.

### Water co-deintercalation during high potential charging

**Fig. 5:**
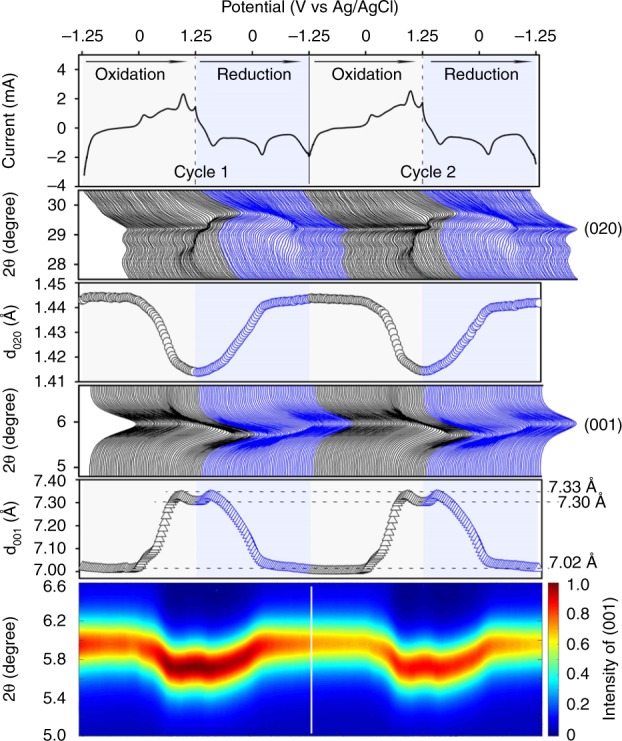
In situ XRD characterization of Na_0.27_MnO_2_. Two CV scans were conducted between −1.25 V and 1.25 V at a scan rate of 0.75 mV s^−1^, showing the changes of d-spacing for (001) basal diffraction plane and (020) Bragg diffraction plane and the contour plot peak variation of (001) plane during the charging (black) and discharging (blue) processes

Supplementary Figure 23 shows in situ XRD measurements conducted along with the CV test at a scan rate of 0.75 mV s^−1^, where diffraction peaks at the 2θ angles of ~5.8° and ~29° can be attributed to (001) basal diffraction peak and (020) Bragg peak, respectively. As shown in Fig. 5, when the potential increased from −1.25 V to 1.25 V (charging), the (001) peak shifted to a lower 2θ angle, corresponding to an increasing interlayer distance of (001) plane (d_001_) because the electrostatic repulsion between the [MnO_6_] layers leads to an increase of interlayer spacing upon the removal of Na-ions, whereas (020) Bragg peak shifted to a higher 2θ angle simultaneously with a decreasing d_020_ due to the increased fraction of Mn^4+^ ions that have a smaller radius than Mn^3+^. During the reduction (from 1.25 V to −1.25 V), (001) and (020) peaks were restored to the original states. Nearly identical behaviors were also found in the second cycle. Na_0.27_MnO_2_ material showed a 4.4% change in the d_001_ spacing of (from 7.33 Å to 7.02 Å) between fully charged and discharged states, more significant than previously reported MnO_2_ birnessite (1.7%) with a potential window of 1.2 V and a low capacity of 36 mAh g^−1^ ^27^. The contour plot in Fig. 5 reflects peak shifts of the (001) basal diffraction peak of Na_0.27_MnO_2_ during charging and discharging process. The observed continuous and reversible peak shifting without a staged structural transformation indicates high structural stability of Na_0.27_MnO_2_ during the charging and discharging processes, which explains the good cycle life of Na_0.27_MnO_2_ materials.

**Fig. 6:**
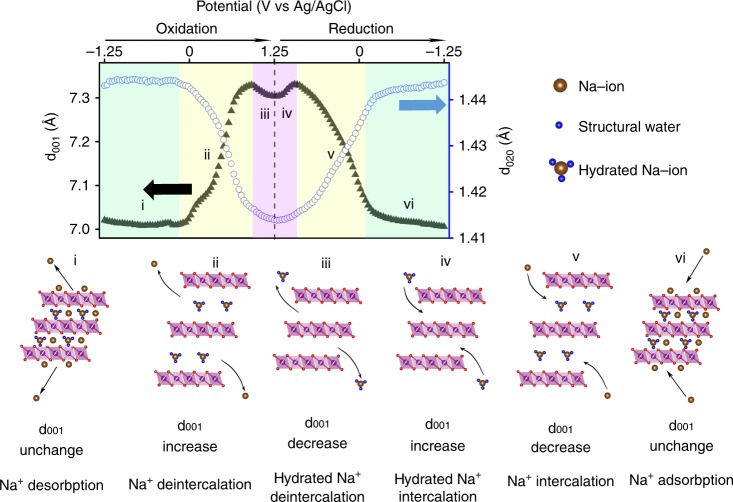
Schematic of Na-ions and water motion during the redox processes. It shows co-deintercalation and co-intercalation of Na-ion and water molecules within the interlayer region of the Na_0.27_MnO_2_ during charging (oxidation) and discharging (reduction) processes

In situ XRD also revealed water trafficking, for the first time, along with Na-ions insertion and extraction during the charging and discharging process. Figure 6 shows that when the potential increased from −1.250 V to −0.106 V, d_001_ and the corresponding electrochemical current remained relatively constant, suggesting a non-Faradaic capacitive charge storage process (e.g., desorption of Na-ions from electrode surface) without extrcting Na-ions from interlayers. When the potential increased from −0.106 V to 0.914 V, d_001_ increased rapidly from 7.02 Å to 7.33 Å, indicating a large amount of Na-ions were extracted from the interlayers, concurrent with the increasing Faradaic current; as the potential continuously increased from 0.914 V to 1.25 V, d_001_ decreased from 7.33 Å to 7.30 Å. Interlayer collpase at higher potential is likely attributed to the removal of structural water along with Na-ion extraction, since a mere Na-ion removal alone would only cause an increase in d_001_. Notably, such compression at high potential only happened in the interlayer distance (along *c*-direction) because d_020_ decreased continuously indicating continued oxidation of Mn^3+^ into Mn^4+^ when the potential increased from −0.106 V to 1.25 V. Although it is possible that when the potential increased from −0.106 V to 0.914 V the extracted Na-ions would also bring structural water out of the interlayer region, it was obvious that weakened electrostatic interaction caused by the Na-ion removal offsets the water removal effect if any, so that the overall interlayer distance still significantly increases. This means that extracted Na-ions at lower anodic potential range (from −0.106 V to 0.914 V) brought much less (or none) hydrated water molecules out of the interlayer region compared with the ones extracted at higher anodic potential (from 0.914 V to 1.25 V). In other words, hydrated Na-ions required higher overpotential to be removed from interlayer region than less hydrated ones. It is possible that O ions (from water) in the solvation shell of the intercalated Na-ions could interact with Mn ions from Mn–O octahedral layer, especially when there are local defects (e.g., anion defect) and Mn cations are under-coordinated as previously reported interaction between V_2_O_5_ and structural water^52^. Such interaction could increase the energy barrier for Na-ion migration at charging process, and thus a higher overpotential will be needed to extract these hydrated Na-ion from host material. It is also possible that water molecules in the solvation shell of Na-ion could form hydrogen bonding in the interlayer region. This argument is also supported by a recent X-ray and neutron total scattering study of the birnessite materials, where hydrogen bonding among the interlayer water molecule and adjacent Mn–O layer oxygen ion was found to play an important role in maintaining the intermediate and long-range stacking of Mn–O octahedral layer^53^. Therefore, hydrogen bonding between structural water could also stabilize the Na-ions inside the interlayer region, thus extraction these hydrated Na-ion out of interlayer region (charging) might become more difficult. Likewise, during the reduction process quite symmetric changes of d_001_ were observed: d_001_ slightly increased from 7.30 Å to 7.33 Å as the potential decreased from 1.25 V to 0.951 V, and then sharply decreased from 7.33 Å to 7.02 Å as the potential continued to decrease from 0.951 V to −0.682 V. This suggests that inserted Na-ions in the higher cathodic potential range (from 1.25 V to 0.951 V) brought more hydrated Na-ions into the interlayer region compared with ones inserted at lower anodic potentials (from 0.998 V to −0.682 V). Congruent with the observation of the water trafficing during the anodic scan, fully hydrated Na-ions preferred to be inserted into the interlayer region than less hydrated ones during the cathodic scan.

In conventional LIB layered oxide cathodes, a decrease in the *c*-lattice is widely observed at higher degrees of delithiation, even when there is no phase transformation^54^. Recent work on a variety of LiNi_1−*x*−*y*_Co_*x*_Mn_*y*_O_2_ (NMC) compounds found the onset of this decrease in the c-lattice when around 50–60% lithium has been removed from the structure with limited dependence on Ni, Co, or Mn content^55^. In those LIB cathodes, strong nickel/cobalt-oxygen covalency is expected to facilitate charge transfer from the O 2p orbitals, decreasing the negative-charge on the oxygen^56^. This effectively weakens electrostatic respulsions between neighboring oxygens across the Li-layer leading to a decrease in the interlayer spacing and thereby a decrease in the c-lattice. Upon relithiation, the c-lattice expands and can symetrically match the collapse observed during delithiation^55,57^. Density functional theory (DFT) calculations have shown this effect occurs for the LiMnO_2_ system and may need to be considered for other Mn(IV)-systems^58^.

**Fig. 7:**
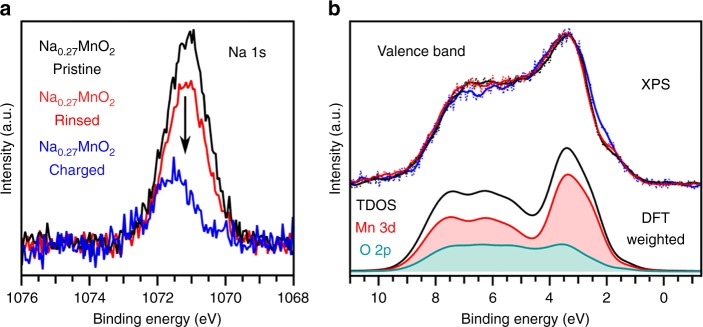
XPS and DTF analysis of Na_0.27_MnO_2_.XPS measurements. **a** Na 1 s region for pristine Na_0.27_MnO_2_ powder, Na_0.27_MnO_2_ powder rinsed in the Na_2_SO_4_ electrolyte, and **b** valence band of Na_0.27_MnO_2_ electrode charged to 1.25 V (vs Ag/AgCl). The valence band XPS spectra are compared with DFT calculations of the TDOS and the Mn 3d and O 2p PDOS for the Na_0.27_MnO_2_ system

We performed XPS measurements for pristine, rinsed, and charged Na_0.27_MnO_2_ in conjunction with DFT calculations, shown in Fig. 7. In this case, the charged Na_0.27_MnO_2_ is binder-free, which allowed for increased sensitivity to the Na_0.27_MnO_2_ compound (Supplementary Fig. 24). When charged to 1.25 V, there is a decrease in the intensity of the Na peak due to the removal of Na-ions from the interlayer (Fig. 7a). The corresponding valence band XPS measurements display only a slight change in the lineshape of the charged electrode compared to the pristine and rinsed samples likely related to the depopulation of states upon the removal of Na-ions. DFT calculations of the total density of states (TDOS) Na_0.27_MnO_2_ system weighted by the X-ray photoionization cross-section^6^ match well with the experimental spectra suggesting these calculations can be used to comment on Mn 3d-O 2p covalency (Fig. 7b). When looking at the weighted O 2p and Mn 3d partial density of states, we find O 2p states contribute at the top of the valence band (0 eV to 2 eV). At 1486 keV, the Mn 3d photoionization cross-section is over four times higher than the O 2p state so that in the unweighted DFT calculations the O 2p orbitals are the dominant contribution from 0 to 2 eV (Supplementary Fig. 25), i. e., there is discernable Mn-O covalency in the Na_0.27_MnO_2_ so that we may have to consider a decrease in the negative-charge on the oxygen with Na-removal. While this highlights the role Mn-O covalency may play in Na-removal, we believe the water trafficking (co-deintercalation with Na-ion) at high potentials remains the primary factor in the observed shrinkage of the interlayer for Na_0.27_MnO_2_ for the following reasons. Firstly, compared to the LIB electrodes with an interlayer spacing < 3 Å, the Na_0.27_MnO_2_ system has an interlayer spacing of 7.1 Å. Thus, the oxygen-oxygen interaction between adjacent [MO_6_] layers in birnessite, which is inversely correlated to the square of the interlayer distance ($\propto \frac{1}{{r^2}}$ where *r* is the distance between neighboring oxygen) is relatively weak compared to the NMC compounds. Moreover, the structural water within the interlayer of Na_0.27_MnO_2_ birnessite provides a “screening” effect that further weakens the O–O interaction, well described by Debye-Huckel theory.

Different from *c*-lattice collapse in charged LIB layered oxide, where the irreversible and large lattice collapse (up to 5%) results in the pulverization of electrode material and impairs their full utilization for Li-ion storage, the reversible co-deintercalation of structual water in Na_0.27_MnO_2_ benefits Na-ion storage. Notably, repetitive insertion and extraction of cations and thus drastic changes of the interlayer distance during prolonged cycling can cause the degradation of the electrode material. Figure 6 showed that as the *c*-lattice contracted, the *b*-lattice continue to decrease (d_001_ and d_020_ decreased). This indicates that the co-deintercalation of water molecules along with Na-ions stabilizes the layered structure against further expansion of the interlayer distance at higher voltages while sustaining an intensive redox process. To the best of our knowledge, this new safety mechanism has never been reported in aqueous energy storage. Notably, previous investigations of intercalation cations (e.g., Na^+^ and Mg^2+^) in aprotic electrolytes in layered materials have reported a similar water-assisted cation insertion, where insertion kinetics can be greatly improved since a water solvation shell partially shields the charge of cations within the cation/water co-intercalation compound^59–63^.

Supplementary Figure 26 shows Na_0.19_MnO_2_ (synthesized via thermal decomposition of NaMnO_4_ materials at 800 °C) had a lower amount of structural water compared with hydrated Na_0.27_MnO_2_ materials, evidenced by its narrower interlayer distance (0.713 nm vs 0.719 nm) as XRD showed (Supplementary Fig. 26). More importantly, the half-cell CP measurements showed that less hydrated Na_0.19_MnO_2_ birnessite had an inferior electrochemical performance to the hydrated Na_0.27_MnO_2_ in term of discharge capacity, rate performance and cycle life (Supplementary Fig. 27). For example, the hydrated Na_0.27_MnO_2_ showed a much higher discharge capacity (138 mAh g^−1^) compared with less hydrated Na_0.19_MnO_2_ (60 mAh g^−1^). Moreover, at a current density of 1 A g^−1^, Na_0.27_MnO_2_ also demonstrated higher discharge capacities and higher capacity retention compared with Na_0.19_MnO_2_ throughout the first 100 charge and discharge cycles (53% vs 29% at 50th cycle and 35% vs 22% at 100th cycle), while maintaining comparable coulombic efficiencies. Thus, it is evident that structural water co-intercalation with Na-ions plays promotional roles in electrochemical performance.

### The disordered structure widens the voltage window

**Fig. 8:**
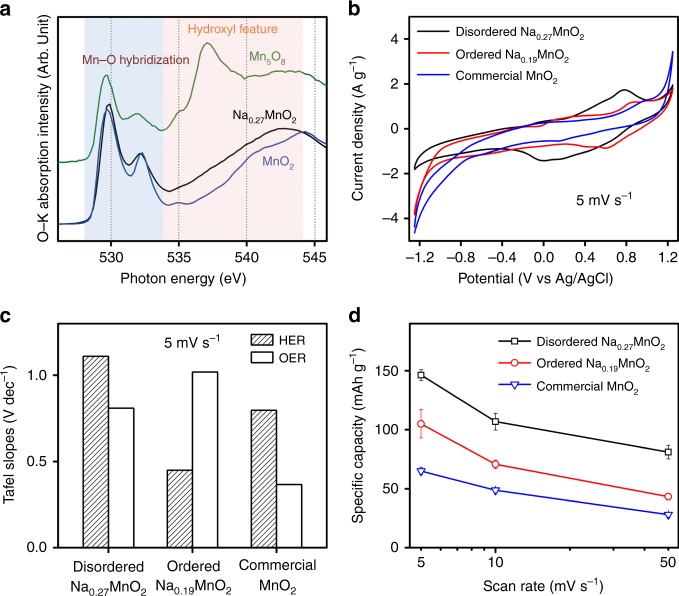
The effects of disorder structure on the potential window. **a** Oxygen K-edge sXAS of electrochemically cycled Na_0.27_MnO_2_, Mn_5_O_8_ and commercial MnO_2_ materials; **b** CV scans of disordered Na_0.27_MnO_2_, ordered Na_0.19_MnO_2_ and commercial MnO_2_ materials at the scan rate of 5 mV s^−1^ in a 2.5 V potential window in half-cell; **c** calculated Tafel slopes of HER and OER at the scan rate of 5 mV s^−1^; **d** Specific capacities at the scan rate of 5, 10 and 50 mV s^−1^

Although Mn_5_O_8_ and Na_0.27_MnO_2_ materials showed a 2.5 V stable voltage window for aqueous Na-ion storage (Supplementary Figs. 10, 28), the mechanisms underlying their high resistance toward HER and OER were completely different. Figure 8a shows that O-K sXAS spectra of Na_0.27_MnO_2_ and Mn_5_O_8_ have similar sharp features below 534 eV from the hybridization between the O 2p band and Mn 3d states. However, only the Mn_5_O_8_ material showed distinct fingerprint water-features at the 535 and 537 eV, indicating the formation of a highly ordered hydroxylated interphase on the surface as we reported previously^48^. To understand role of disordered structure in mitigating the water decomposition, we synthesized ordered Na_0.19_MnO_2_ birnessite via thermal decomposition of NaMnO_4_ materials at 800 °C and conducted the Tafel analysis for hydrogen evolution reaction (HER) and oxygen evolution reaction (OER) on disordered Na_0.27_MnO_2_, ordered Na_0.19_MnO_2_ and commercial MnO_2_ materials in a 0.1 M Na_2_SO_4_ electrolyte. XRD and X-ray PDF were conducted to compare the structural difference of three materials (Supplementary Fig. 29–30, Supplementary Tables 8–10), showing that disordered Na_0.27_MnO_2_ had a triclinic (C-1) birnessite structure. Though disordered Na_0.27_MnO_2_ and ordered Na_0.19_MnO_2_ both show the birnessite layered structure, Na_0.27_MnO_2_ has a smaller crystalline size and a shorter coherence length (Supplementary Fig. 29b) compared to Na_0.19_MnO_2_. On the other hand, commercial MnO_2_ showed a highly crystalline and ordered β-MnO_2_ phase. Supplementary Figure 30 demonstrated the difference of local structures between disordered Na_0.27_MnO_2_ and ordered Na_0.19_MnO_2_ birnessite and commercial MnO_2_, where the former showed a more disordered lattice structure.

Figure 8b shows the CVs of disordered Na_0.27_MnO_2_, ordered Na_0.19_MnO_2_ and commercial β-MnO_2_ and a scan rate of 5 mV s^−1^ (CVs tested at other scan rates are shown in Supplementary Fig. 31). Though all three materials were tested in a 2.5 V potential window, ordered Na_0.19_MnO_2_ and commercial β-MnO_2_ materials displayed obvious gas evolution features at lower and higher potential ranges. The Tafel analysis showed that disordered Na_0.27_MnO_2_ displayed a much weaker HER current at potentials up to −1.25 V (overpotential of 0.63 V towards HER) and higher Tafel slopes at various scan rates (Fig. 8c, Supplementary Fig. 31), suggesting sluggish HER kinetics. It is notable that three materials were inactive towards OER even at a potential of 1.25 V (overpotential of 0.63 V towards OER), however, only disordered Na_0.27_MnO_2_ has high overpotential for both HER and OER, suggesting that the disordered nature leads to high resistance to the gas evolution reactions, thus a kinetically stable potential window of 2.5 V. Although ordered Na_0.19_MnO_2_ and commercial β-MnO_2_ materials showed a great capacity enhancement at a lower potential range close to −1.25 V, probably benefiting from hydrogen insertion, disordered Na_0.27_MnO_2_ still showed much superior capacities at all tested scan rates. The inferior capacities from ordered Na_0.19_MnO_2_ and commercial β-MnO_2_ might be due to the parasitic gas evolution reactions especially HER that degraded the electrode and causes capacity loss at prolonged cycles. Previous DFT calculations demonstrated that the thermodynamically unstable edge sites of the layered transition-metal dichalcogenides nanocrystals were catalytically active for HER^47^. Previous studies showed that HER current was proportional to the length of edges rather than the coverage area of catalysts^64,65^, but these catalytically active sites were located on the thermodynamically unstable planes (edges of the layers), which are difficult to be exposed preferentially^66,67^. In this study, disordered MnO_2_ layered structures have highly exposed (001) planes that are thermodynamically stable plane but catalytically inert, while the ordered Na_0.19_MnO_2_ birnessite possesses a large grain size with a long coherent length, therefore the edges of the layers that are more active toward gas evolution are likely exposed.

## Discussion

In this work, we have integrated detailed structural analysis with electrochemical measurements to understand the observed high capacity and good structural stability found in the disordered and Na-rich Na_0.27_MnO_2_ birnessite layered materials. In situ XRD has revealed the role of water co-deintercalation in mitigating interlayer expansion during the high potential charging. Investigations of solvent co-intercalation properties in other layered materials will be useful in designing high capacity rechargeable aqueous batteries. In addition to water trafficking, our results also manifest the promotional effects of the disordered structure on aqueous Na-ion storage: disordered Na_0.27_MnO_2_ structure allows continuous and smooth structural evolution during the charging and discharging processes without phase transitions and possesses highly exposed (001) planes with low density of active edge sites for gas evolution reactions, and thus yields a large aqueous Na-ion storage capacity and long cycling life. The reported results have provided the insight underlying the promotional roles of structural water and disordered electrode structure for aqueous Na-ion storage. Especially, the structural water trafficking at high potential charging discovered here may provide a fundamental leap forward in current understanding how water molecules stabilize the electrode structure during redox processes.

## Methods

### Material synthesis

Mn_3_O_4_ nanoparticles were first synthesized via a solution phase method. In a typical synthesis, MnCl_2_∙4H_2_O (0.7 g, Alfa Aesar, 99% metals basis) was fully dissolved by deionized water (140 mL, 18.2 MΩ; Millipore, Inc.) in a 500 mL flask under vigorous stirring at room temperature. The aqueous solution of NaOH (Alfa Aesar, 99.98% metals basis) with a concentration of 0.123 g mL^−1^ was injected at a rate of 0.167 mL min^−1^ for 50 min using an automatic syringe (HSW Inc.). After injection, the mixture continuously reacted for another 30 min till dark brown precipitate was formed. The resulting product was separated by centrifuging and then washed by deionized water and ethanol consecutively. The obtained products (Mn_3_O_4_ nanoparticles) were finally vacuum-dried. In the synthesis of sodium-manganese oxides, NaOH (Alfa Aesar, 99.99% metals basis) and 100 mg Mn_3_O_4_ nanoparticles were grounded using a mortar and pestle with the molar ratios of 0.5, 1, 1.5, 2, and 4, respectively. The resulting mixture of NaOH and Mn_3_O_4_ was heated in a tube furnace (Thermal Scientific, Inc.) in open air at 270 °C for 6 h. The obtained solids were thoroughly washed with deionized water to remove the possible NaOH residual and vacuum-dried for overnight. Ordered Na_0.19_MnO_2_ birnessite was synthesized via thermal decomposition of NaMnO_4_ at 800 °C for 12 h, and then washed by deionized water and ethanol, and dried in vacuum. The MnO_2_ birnessite with low sodium concentration Na_0.13_MnO_2_ was synthesized via a wet chemistry method. Aqueous MnCl_2_ (5 mg mL^−1^) precursor was injected into 20 mL NaOH solution with a concentration of 5.7 mg mL^−1^ at the rate of 0.167 mL min^−1^ for 1 h, and the obtained product was vacuum-dried after washed by deionized water and ethanol. And then the solids were annealed in air at 270 °C for 6 h.

### Material characterizations

Inductively coupled plasma mass spectrometry (ICP-MS) was used to identify the elemental ratios of materials. Sample aliquots were digested in mixed concentrated HCl-HNO_3_ solution and then transferred into HNO_3_ solution for dilution in 2% HNO_3_ and introduction into the Nu instruments AttoM high resolution ICP-MS. Standards of known concentrations were used to correct for drift and within-instrument elemental fractionation. Triplicate runs of each sample allowed for the determination of the precision of each sample. Energy dispersive X-ray spectroscopy (EDXS) was conducted for elemental analysis by an Amray 3300FE field emission SEM with a PGT Imix-PC microanalysis system at the University of New Hampshire. Thermogravimetric analysis (TGA) was measured on a Mettler-Toledo instrument at the University of New Hampshire. Regular transmission electron microscopy (TEM) images were collected on Zeiss/LEO 922 Omega TEM at the University of New Hampshire. X-ray photoelectron spectroscopy (XPS) was measured using Thermo Scientific K-Alpha instrument at Harvard University.

### Half-cell test

Cyclic voltammetry (CV) measurements of sodium-manganese oxide were conducted using a three-electrode half-cell powered by CHI 660d single channel electrochemical workstation. The three-electrode system contained a glassy carbon rotating disc electrode (Pine Instrument) as the working electrode, platinum wire and silver-silver chloride (Ag/AgCl) electrode as counter and reference electrodes, respectively. The ink material was prepared by grinding mixture of 7 mg sodium-manganese oxide and 3 mg carbon black (Alfa Aesar, > 99.9%). The resulting mixture was mixed with deionized water to make an ink solution of 0.5 mg mL^−1^. The resulting solution was subsequently sonicated until the materials were homogeneously dispersed. In a typical half-cell measurement, 10 μL suspension containing 3.5 μg sodium-manganese oxide and 1.5 μg carbon black was drop-cast onto the glassy carbon disc electrode (0.5 cm in diameter) and vacuum-dried. The CV measurements of electrodes were conducted in a 250 mL flat-bottom flask containing 100 mL argon-purged Na_2_SO_4_ aqueous electrolyte (0.1 M) at a rotating rate of 500 rpm. The CV data were obtained within an applied potential range from −1.25 V to 1.25 V (vs Ag/AgCl) for three cycles, and the third CV cycle was used for the calculation of storage capacity. The CP measurements of Na_0.27_MnO_2_ in a three-electrode half-cell was also conducted at the currrent densities from 0.3 A g^−1^ to 2.0 A g^−1^ with potential from −0.75 V to 1.25 V (vs Ag/AgCl). Na_0.27_MnO_2_ was charged to 1.25 V (vs Ag/AgCl) from open circuit voltage in 0.5 M Na_2_SO_4_ solution with a scan rate of 1 mV s^−1^ by using CV in half-cell, and then discharged to −1.25 V. The charged and dischaged samples were washed and colloected for ex-situ XPS measurements.

### Diffusivity measurements

The diffusivity measurements were tested in a typical half-cell setting as described above, except 40 ug active materials sodium-manganese oxides was loaded on working electrode and 0.25 M Na_2_SO_4_ was used as the electrolyte. A constant negative current pulse of 1 uA was first applied to the working electrode and was held for 15 s to discharge the electrode from the open circuit potential. After that, the working electrode was relaxed and potential changes were collected for another 1000 s.

### Potential of zero charge

The potential of zero charge (pzc) of Na_0.27_MnO_2_ materials was estimated using an open circuit voltage (OCV) measurement using a three-electrode cell in a 0.1 M Na_2_SO_4_ electrolyte. Supplementary Figure 32 shows that the initial potential of the electrode was 0.47 V (vs Ag/AgCl), slowly increased to a rather stable value of 0.50 V after 2 h without an external field. In this context, pzc of Na_0.27_MnO_2_ materials should be around 0.5 V vs. Ag/AgCl. As a higher (or lower) external potential is applied, the materials will be oxidized (or reduced) by Na-ion extraction (or insertion).

### Full-cell test

Symmetric two-electrode full-cells with Na_0.27_MnO_2_ electrodes were assembled and measured to characterize the energy/power performance and the long cycle stability as well. Electrodes were made by drop casting the slurry containing ~5 mg Na_0.27_MnO_2_ and 1.25 mg carbon black as a mass ratio of 4:1 on Toray carbon paper (E-Tek, Inc., 1.5 cm in diameter). The resulting electrodes were weighed with an accurate mass loading of active material after vacuum-dried overnight. Two symmetric electrodes were separated by cellulose-based filter paper (Whatman), and 150 µL Na_2_SO_4_ aqueous solution (1 M) was used as the electrolyte. The cell stack of electrodes and separator were tightened by stainless plate and compression spring to ensure good electrical contact and then assembled in the split button-cells (model: EQ-STC, MTI Corp.). Galvanostatic charge and discharge measurements of symmetric full-cells were conducted on the battery analyzer (model: B-TG, Arbin Instruments) within 2.5 V potential window for 5000 cycles at the constant current densities of 1, 2, 5, and 10 A g^−1^. All the electrochemical calculations are provided in the supporting information. Toray paper was used as current collect for symmetric full-cell measurements and stable in 2.5 V without obvious generation of hydrogen (Supplementary Fig. 17).

### Electrochemical impedance measurements

Electrochemical impedance spectroscopy (EIS) measurements were conducted after each charge and discharge cycle in full cell at the open circuit potential with a perturbation of 5 mV and frequency range from 0.1 to 100 kHz, and the Nyquist plots were collected.

### X-ray and neutron scattering characterizations

X-ray diffraction measurements were conducted at Beamline 17-BM-B at the Advanced Photon Source at the Argonne National Laboratory with a wavelength of λ = 0.72768 Å. In situ XRD of electrochemical half-cell measurements were conducted in a home-made cell consisted of thin carbon paper (E-Tek, Inc.) as working electrode, platinum wire and micro Ag/AgCl electrode as counter and reference electrodes, respectively. The Na_2_SO_4_ aqueous electrolyte (1 M) was used as the electrolyte. The suspension of a mixture of Na_0.27_MnO_2_ and carbon black was drop-cast on the thin carbon paper and then dried naturally in air. The cellulose-based filter paper was used as a separator. The cell was then assembled for X-ray measurements. In situ XRD tests were performed during CV scans from −1.25 V to 1.25 V (vs Ag/AgCl) at the scan rates of 0.75 mV s^−1^. GSAS-II software was used to analyze the structural changes during the charge and discharge processes. The X-ray total scattering experiment was also conducted at Beamline 17-BM-B using a wavelength of 0.24116 Å. The total neutron scattering experiment was conducted at the Nanoscale-Ordered Materials Diffractometer (NOMAD) beamline at Spallation Neutron Source at Oak Ridge National Laboratory. The atmoic Pair Distribution Function (PDF) analysis was conducted using PDFgui software.

### Theoretical methods

Density functional theory (DFT) calculations were performed using the WIEN2k software package, which uses full potential and linearized augmented plane waves with local orbitals (LAPW + lo) to self consistently solve the Kohn-Sham equations^61^. The generalized gradient approximation of Perdew, Burke, and Ernzerhof (GGA-PBE) was used for the exchange and correlation energies^62^. The plane-wave cutoff parameters R_MT_K_max_ and G_max_ were selected as 6.5 and 12, respectively, and the cutoff energy was −6.0 Ry. The k-points of the cell was $\left( {1 \times 12 \times 4} \right)$ and for the NaMnO_2_·H_2_O.

## Supplementary information

Supplementary Information

## Data Availability

The authors declare that the data supporting the findings of this study are available within the paper and its supplementary information files.
