# Supplementary material for: Structural water and disordered structure promote aqueous sodium-ion energy storage in sodium-birnessite
Source: Nat Commun. 2019 Oct 31;10:4975. doi: 10.1038/s41467-019-12939-3 (PMC6823464; doi:10.1038/s41467-019-12939-3)
Supplement: Supplementary file 1 — Supplementary Information [file 41467_2019_12939_MOESM1_ESM.pdf]

Supplementary Information

Structural Water and Disordered Structure Promote Aqueous Sodium-Ion Energy Storage in Sodium-Birnessite

Shan et al.

Supplementary Figures

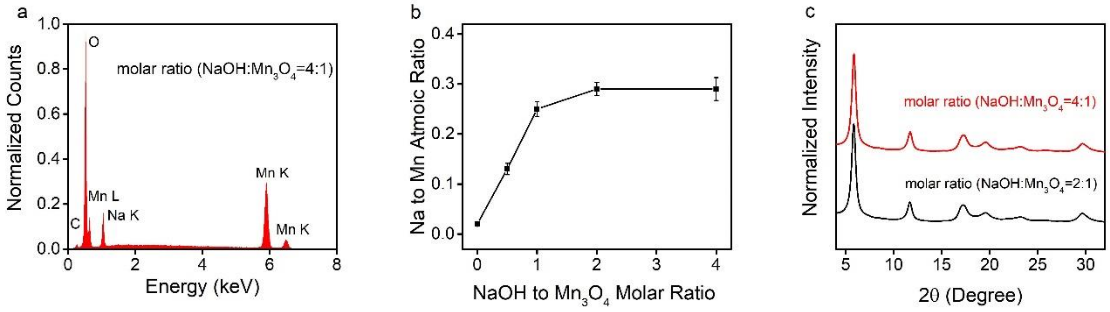

Supplementary Figure 1. EDXS and XRD of sodium-manganese oxides. (a) EDXS characterization of $Na_{\delta}MnO_x$ material obtained by the thermal solid-state reaction of NaOH and Mn_3O_4 at a molar ratio of 4:1; (b) the summarized atomic ratios of sodium to manganese for various $Na_{\delta}MnO_x$ materials; and (c) XRD patterns of $Na_{0.27}MnO_x$ materials syntheized via solid state reaction of NaOH:Mn_3O_4 as molar ratios of 2:1 and 4:1, indicating the forming of a stable $Na_{0.27}MnO_2$ material.

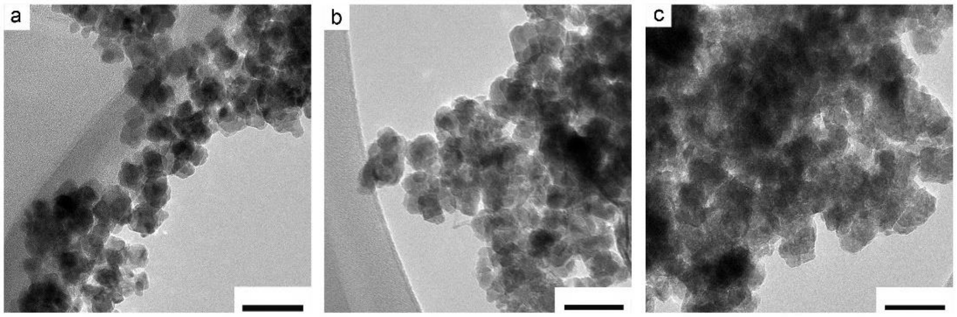

Supplementary Figure 2. TEM images of sodium-manganese oxides. TEM images of the $Na_{\delta}MnO_x$ materials with (a) $Na_{0.01}MnO_x$, (b)$Na_{0.10}MnO_x$, (c) $Na_{0.17}MnO_x$, scale bar, 50nm.

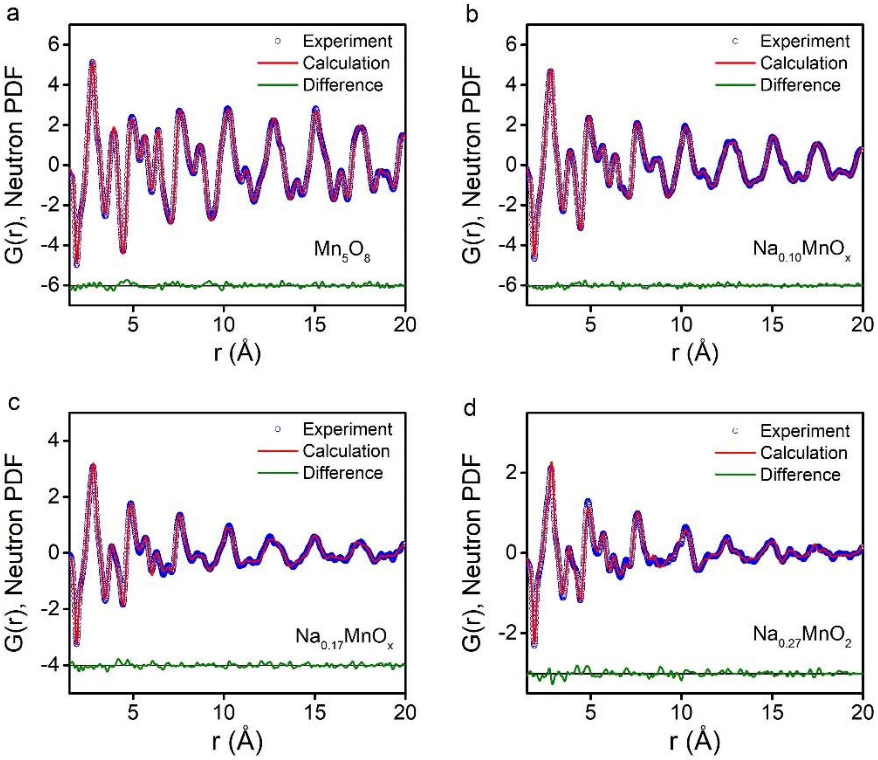

Supplementary Figure 3. Neutron PDF analysis of Mn_5O_8 and $Na_\delta MnO_x$ materials (δ = 0.10, 0.17, 0.27).

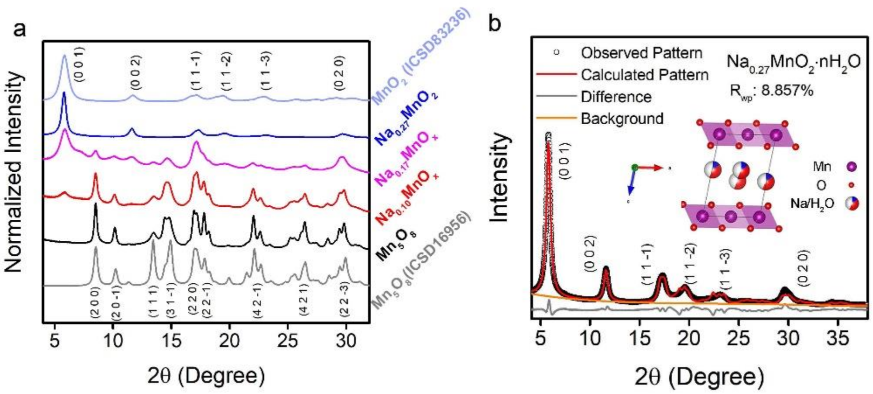

Supplementary Figure 4. XRD patterns of the $Na_{\delta}MnO_x$ materials comapred with Mn_5O_8 and MnO_2 standards indicating the structural evolution from Mn_5O_8 to MnO_2 as sodium concentrations increases, and the Rietveld refinementof the XRD pattern of $Na_{0.27}MnO_2$.

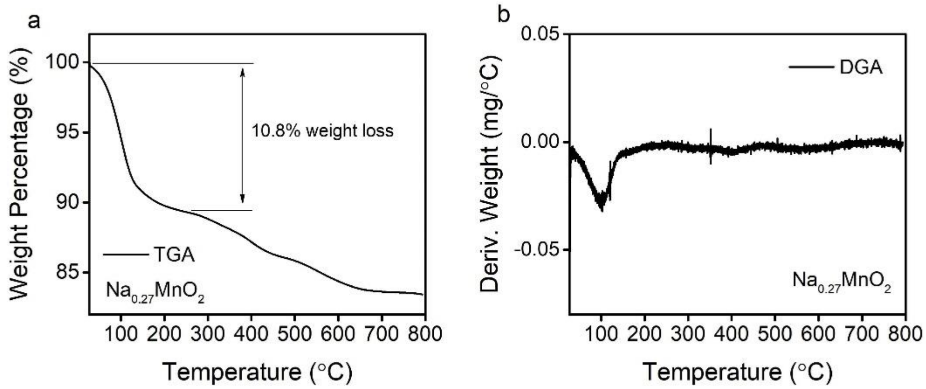

Supplementary Figure 5. TGA and DGA curves of $Na_{0.27}MnO_2$ material with the determination of the amount of structural water (weakly bonded water is neglected since the sample was annealed in air at 270 °C for 6 hours before TGA measurements).

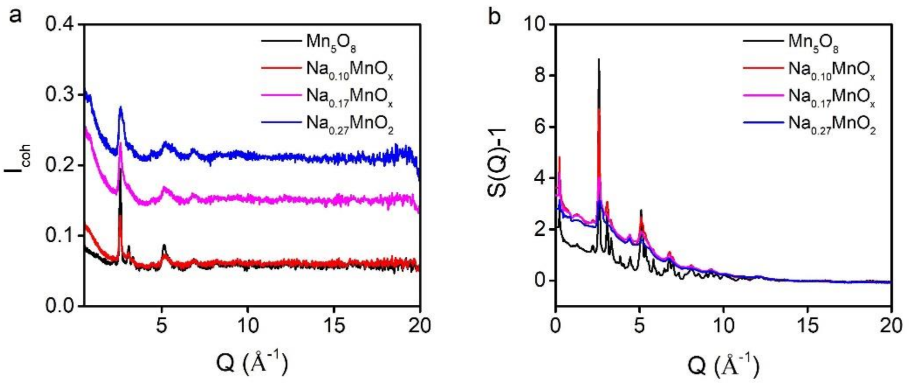

Supplementary Figure 6. Neutron PDF data reduction. (a) Coherent scattering intensity and (b) [S(Q)−1] as functions of Q ($Q=4\pi(\sin\theta)/\lambda$, where λ is the wavelength of neutron and θ is the diffraction angle) of $Na_\delta MnO_x$ samples after the correction of background and standards.

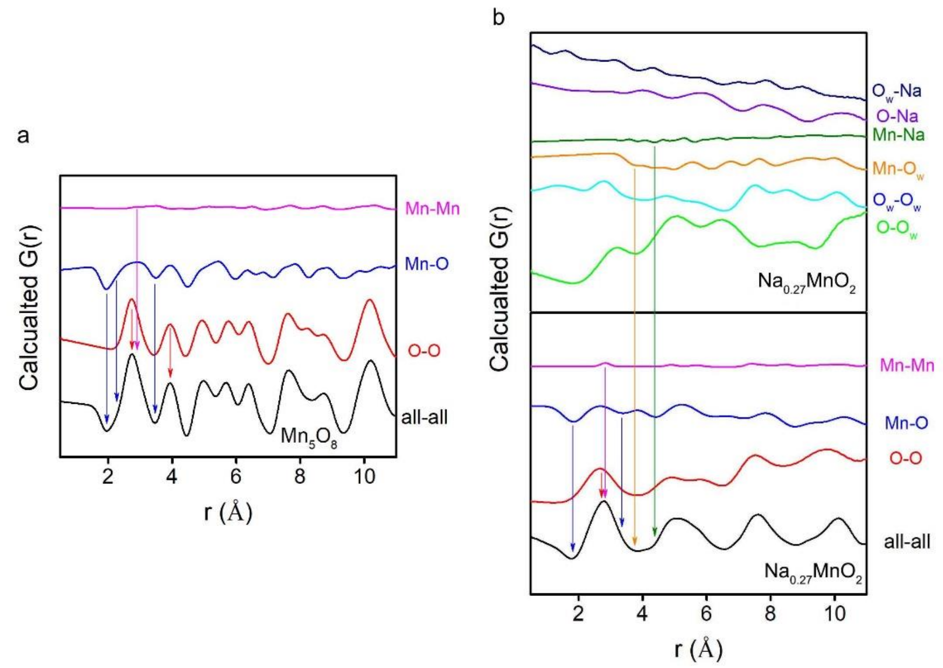

Supplementary Figure 7. Atomic pair contribution analysis. (a) Pair contributions from O-O, Mn-O, Mn-Mn in Mn_5O_8; (b) pair contributions of O-O, Mn-O, Mn-Mn in $Na_{0.27}MnO_2$ and pair contributions from O-O_w, O_w-O_w, Mn-O_w, Mn-Na, O-Na, O_w-Na in $Na_{0.27}MnO_2$. Note: O_w is interlayer O from water and O_+ is the O from Mn-O polyhedra. The interaction of O_w with other atoms was calculated by replacing the O_w with flourine (F) atom due to an identical singal towards neutron scattering in order to differentiate from O in Mn-O polyhedra.

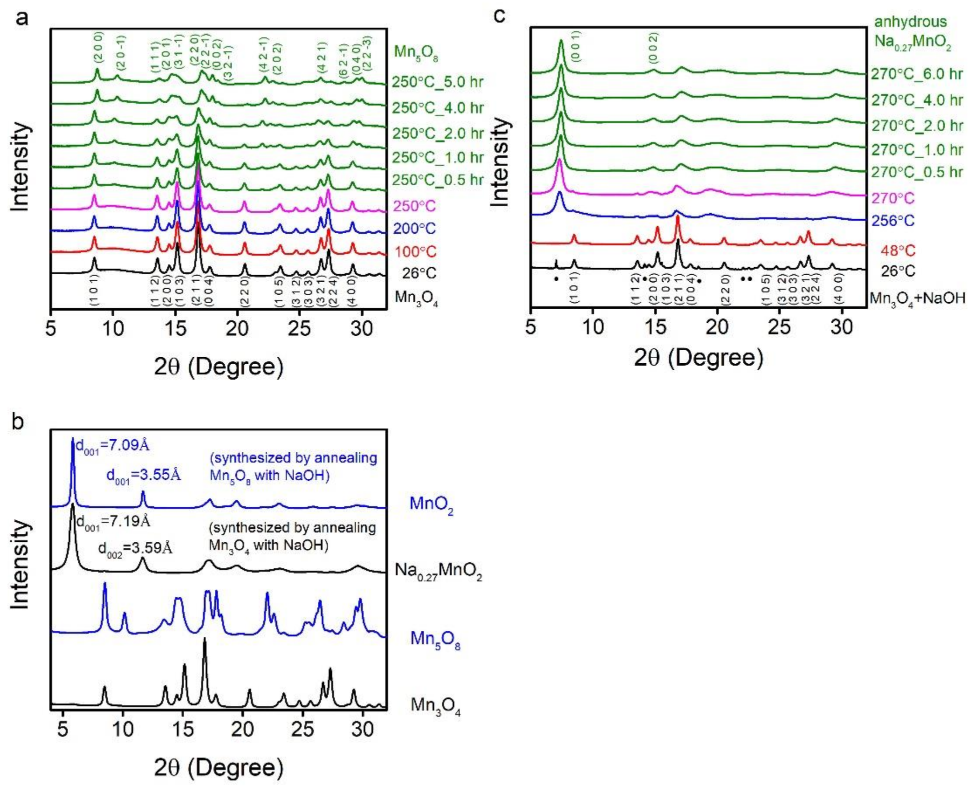

Supplementary Figure 8. *In-situ* XRD during heating. (a) *In-situ* XRD during heating Mn_3O_4 in air forming Mn_5O_8; (b) the *ex-situ* XRD patterns of Mn_3O_4, Mn_5O_8, $Na_{0.27}MnO_2$ (obtained by heating the mixture of NaOH and Mn_3O_4 at a molar ratio of 2:1) and MnO_2 (obtained by heating the mixture of NaOH and Mn_5O_8 at a molar ratio of 2:1), (c) *in-situ* XRD during heating the mixture of NaOH and Mn_3O_4 at a molar ratio of 2:1.

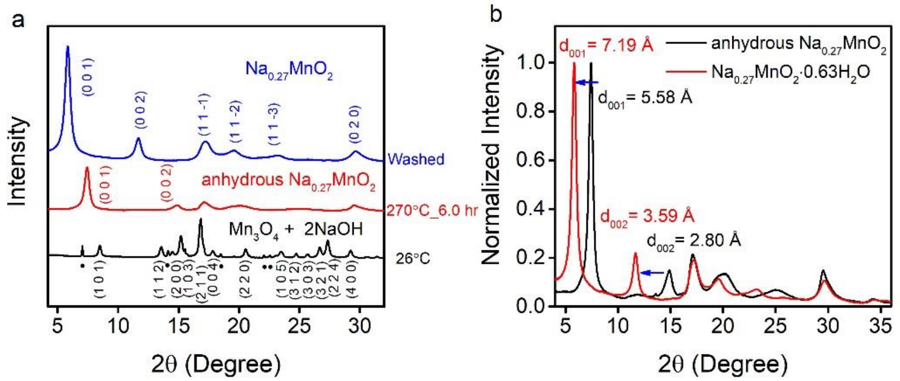

Supplementary Figure 9. XRD characterizations of $Na_{0.27}MnO_x$ materials with the thermal solid-state reacted $Na_{0.27}MnO_2$ and washed $Na_{0.27}MnO_2$ (the diffraction peaks from Mn_3O_4 are indexed in black and the black solid dot from NaOH, the resulting heated anhydrous $Na_{0.27}MnO_2$ and washed $Na_{0.27}MnO_2$ are labelled as well).

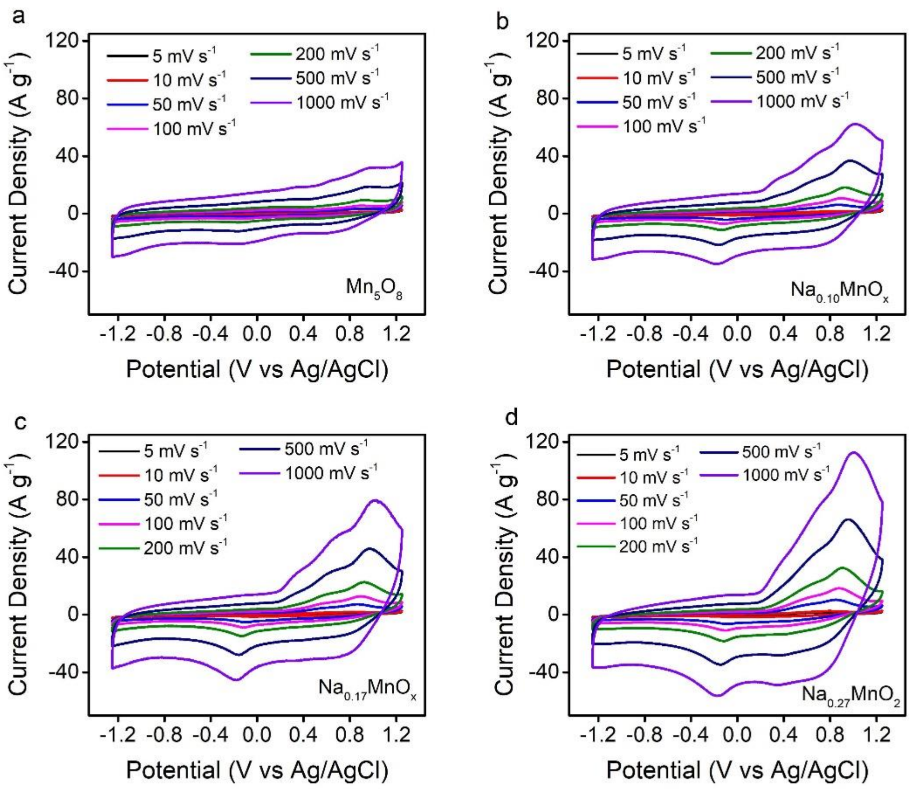

Supplementary Figure 10. CV measuremetns of sodium-manganese oxides in half-cell. Electrochemcial CV meausrements in the three-electrode half-cells. Cyclic Voltammetry (CV) scans of (a) Mn_5O_8, (b) $Na_{0.10}MnO_x$, (c) $Na_{0.17}MnO_x$, and (d) $Na_{0.27}MnO_2$ between −1.25 V to 1.25 V (vs Ag/AgCl) in 0.1 M Na_2SO_4 electrolyte at the scan rates from 5 to 1000 mV s^{-1}.

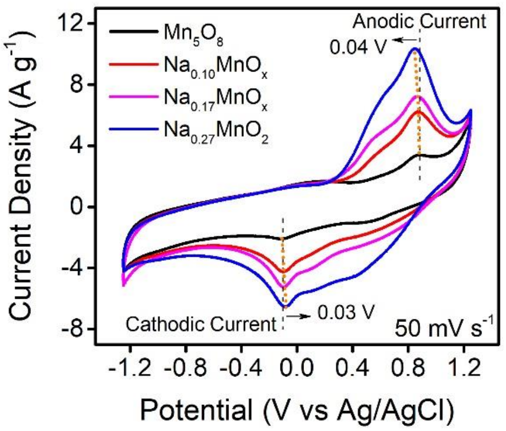

Supplementary Figure 11. CVs of sodium-manganese oxides at the scan rate of 50 mV s^{-1}, showing the anodic peak of $Na_{0.27}MnO_2$ shifted to a lower potential and the cathodic peak shifed to a higher potential compared with those of other materials as the Na concentration increased.

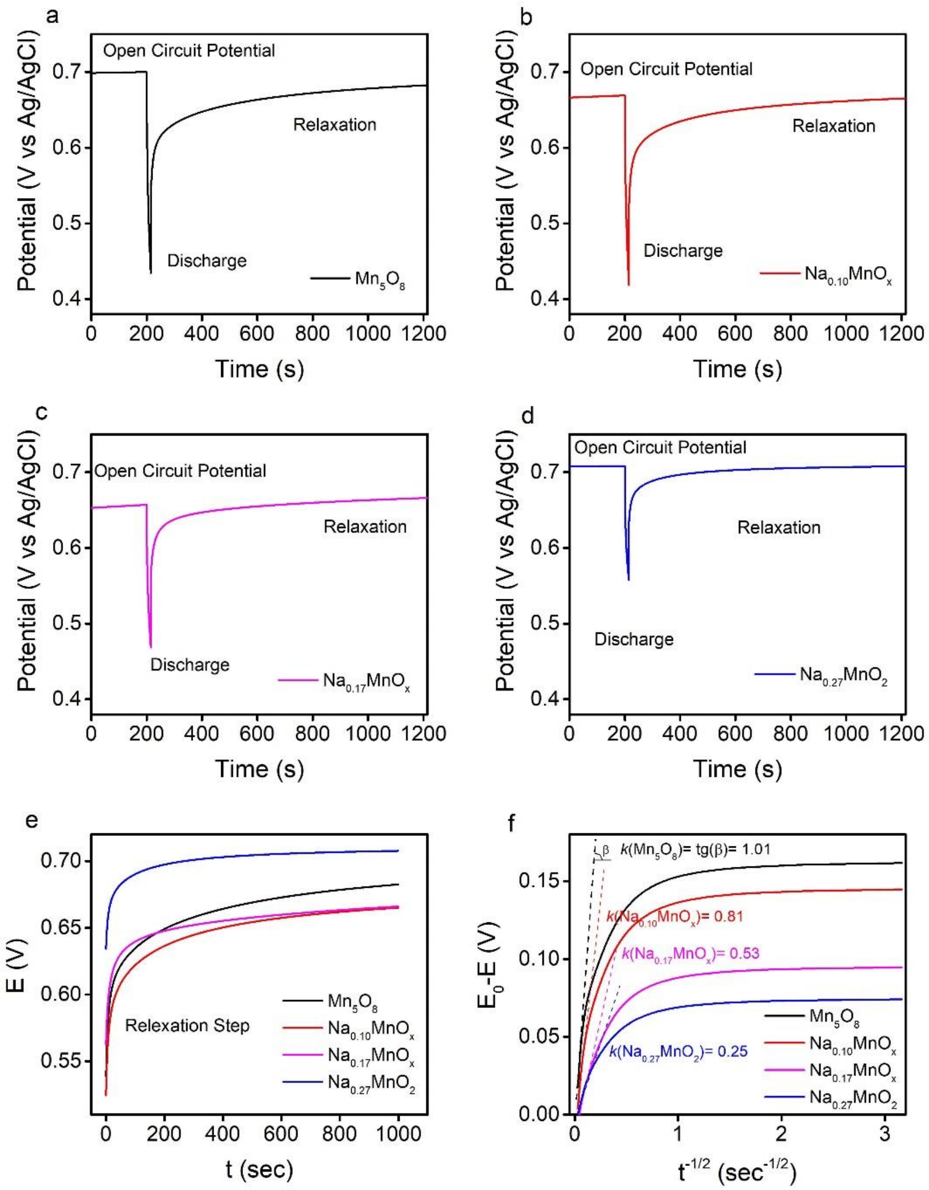

Supplementary Figure 12. Diffusivity tests. Diffusivity measurements of $Na_{\delta}MnO_x$ materials with (a) Mn_5O_8, (b) $Na_{0.10}MnO_x$, (c) $Na_{0.17}MnO_x$, (d) $Na_{0.27}MnO_2$, (e) the relaxation steps of $Na_{\delta}MnO_x$ materials and (f) (E_0-E) as a function of $(1/t^{1/2})$ curves for the slope calculations, where E_0 is the open circuit voltage.

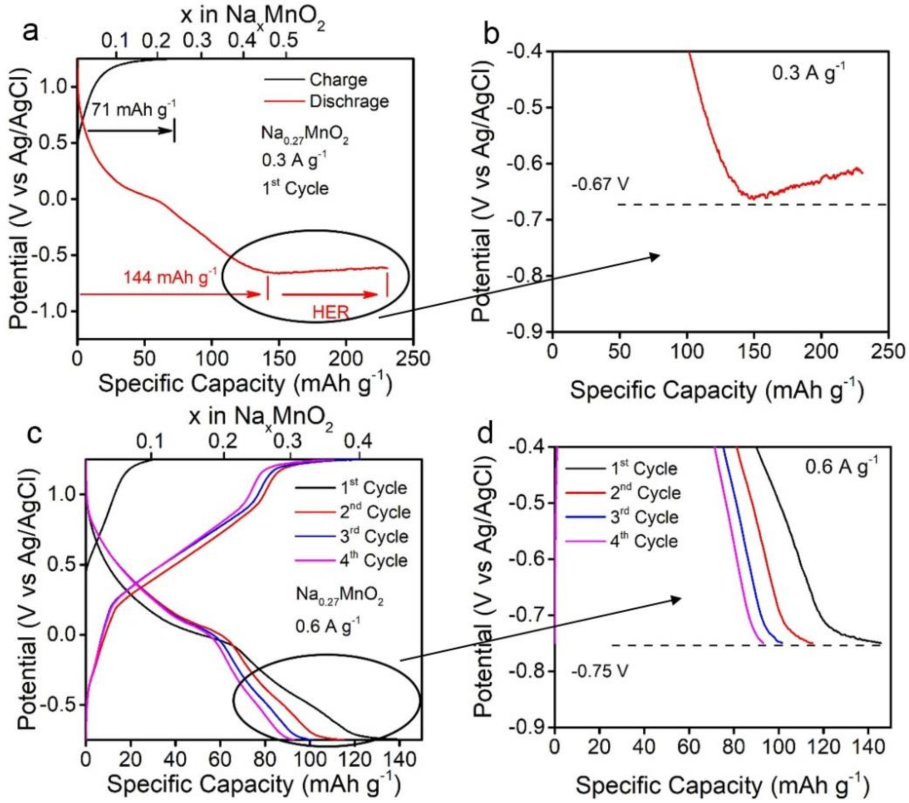

Supplementary Figure 13. CP measurements of $Na_{0.27}MnO_2$ at 0.3 A g^{-1} and 0.6 A g^{-1}. (a,b) CP tests of the charge and discharge cycles at a current density of 0.3 A g^{-1}, where capacity around 144 mAh g^{-1} could be attributed to Na-ion storage, while addition charge transfer could be attributed to hydrogen evolution reaction (HER); (c,d) Four charge and discharge cycles of CP tests conducted at a current density of 0.6 A g^{-1}.

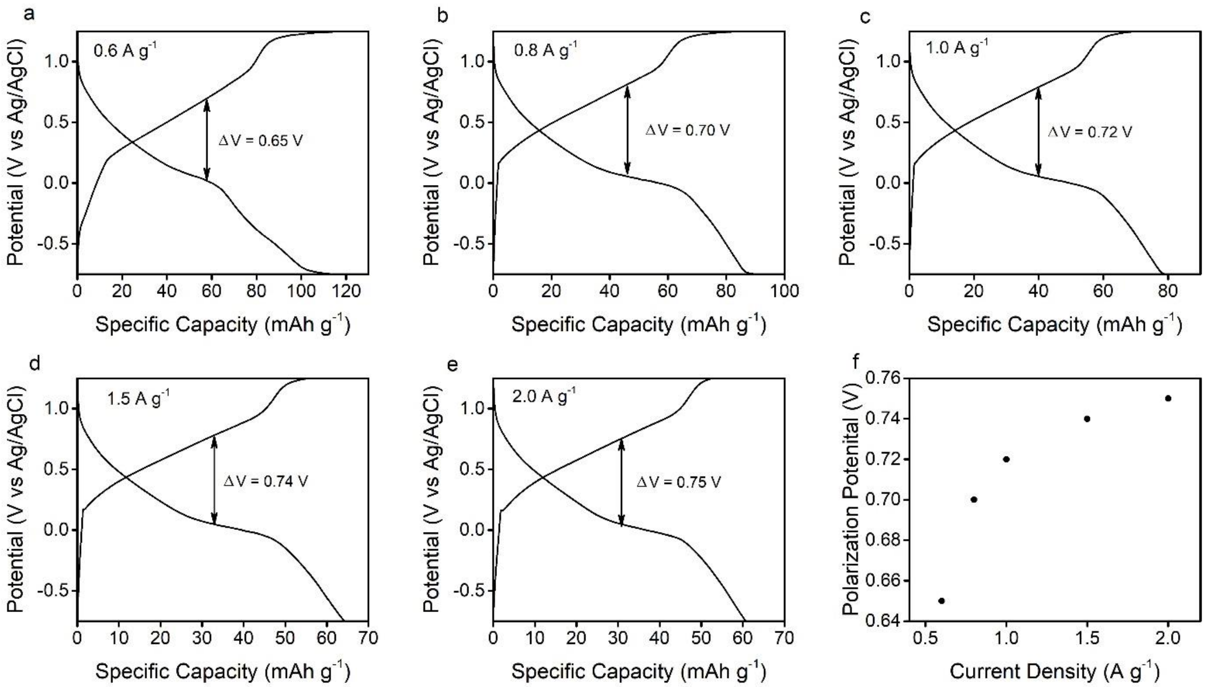

Supplementary Figure 14. The charge and discharge potential differences at the midpoint of the capacity of $Na_{0.27}MnO_2$ at the current densities from 0.6 A g^{-1} to 2.0 A g^{-1}.

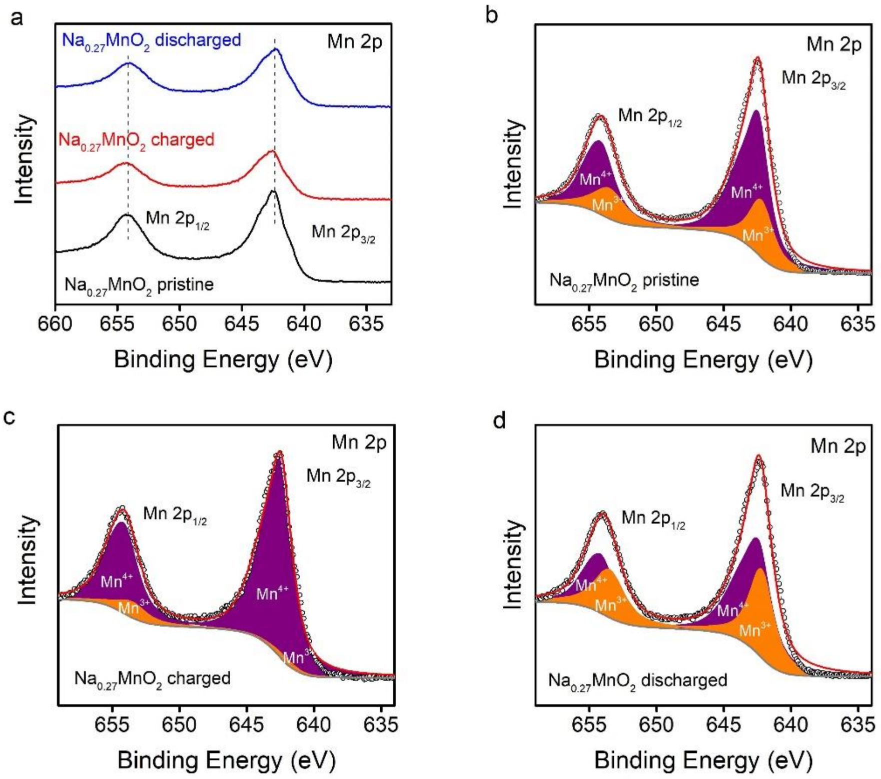

Supplementary Figure 15. XPS data of $Na_{0.27}MnO_2$ at various oxidation states. Ex situ XPS of $Na_{0.27}MnO_2$ with Mn 2p spectra with (c) comparison of pristine, charged and discharged samples; deconvolution analysis of (d) pristine $Na_{0.27}MnO_2$, (e) charged $Na_{0.27}MnO_2$ and (f) discharged $Na_{0.27}MnO_2$.

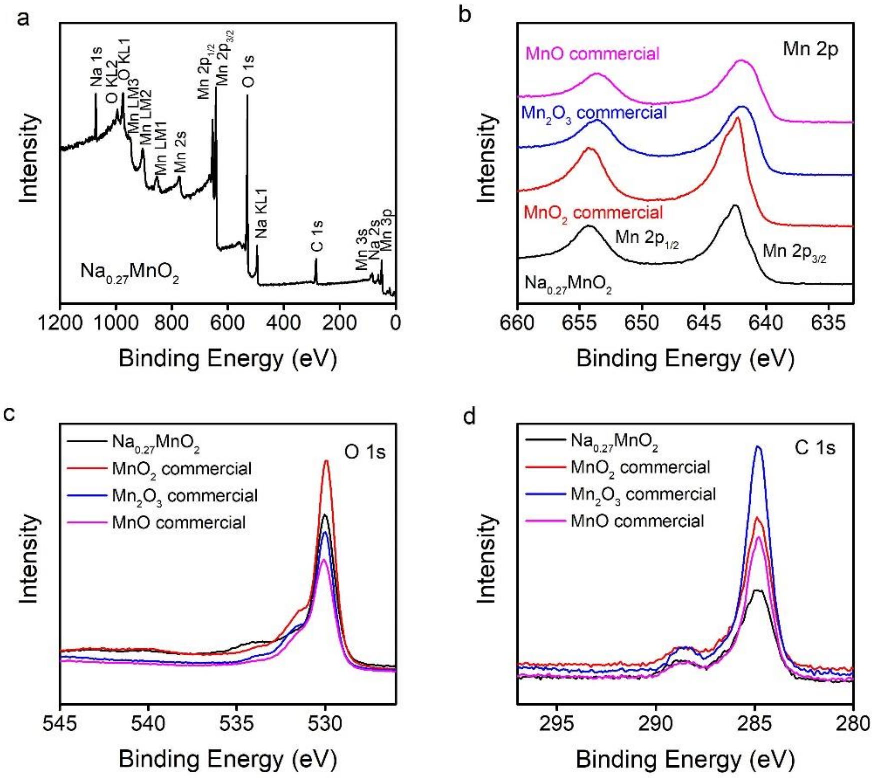

Supplementary Figure 16. XPS data of $Na_{0.27}MnO_2$ compared to other commerical manganese oxides. XPS spectra of $Na_{0.27}MnO_2$ with (a) survey spetrum; (b) Mn 2p compared to other commercial manganese oxides MnO, Mn_2O_3 and MnO; (c) O 1s and (d) C 1s corrected with the binding energy of 284.8 eV.

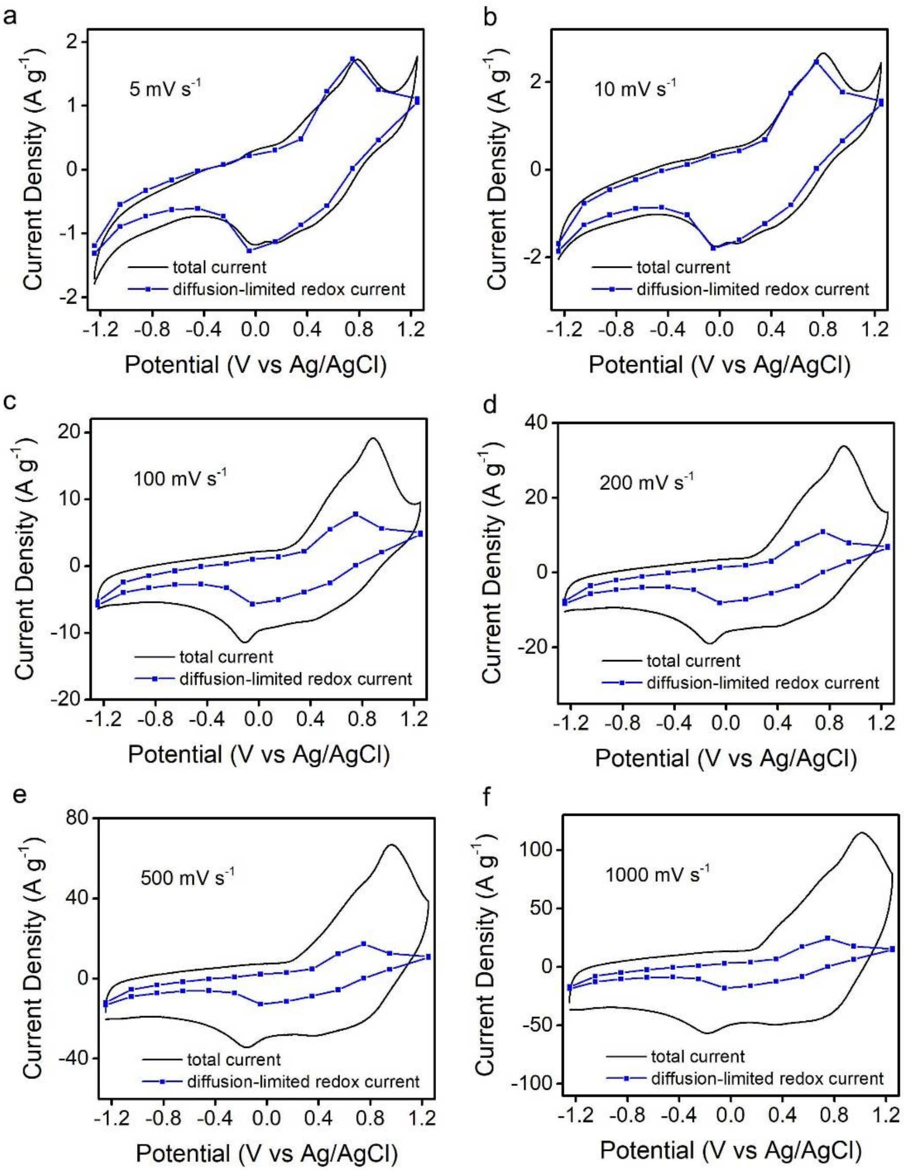

Supplementary Figure 17. Electro-kinetic analysis of $Na_{0.27}MnO_2$ materials in half-cells. CV curves from experimental total current and calculated diffusion-limited redox current at each fixed potentials at the scan rates of 5, 10, 100, 200, 500 and 1000 mV s^{-1}, the percentages of diffusion-limited redox and surface-controlled capacitive contributions are provided.

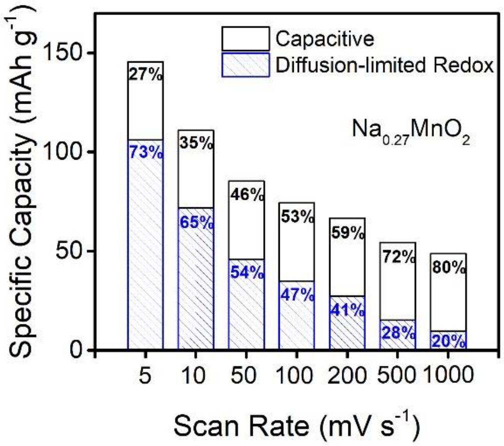

Supplementary Figure 18. Diffusion–limited redox current and capacitive current contribution of $Na_{0.27}MnO_2$

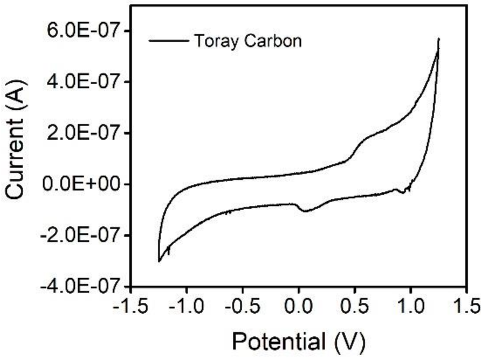

Supplementary Figure 19. Toray carbon paper tested in two electrode symmetric full cell.

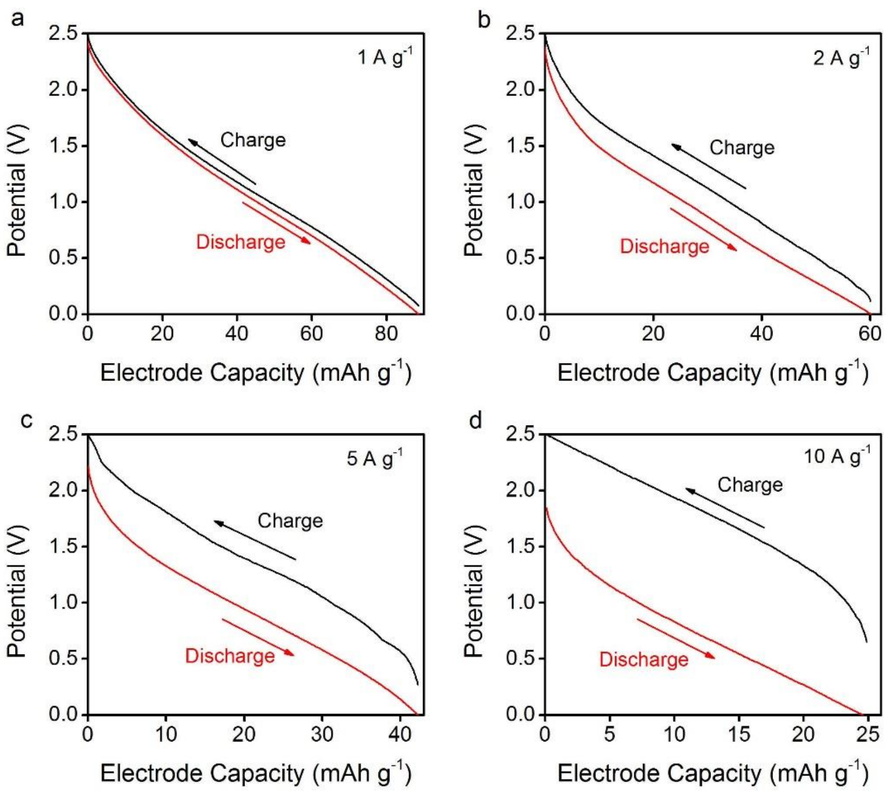

Supplementary Figure 20. Charge and discharge curves of $Na_{0.27}MnO_2$ in full-cell. Electrode capacities of $Na_{0.27}MnO_2$ as a function of voltage at the current denties of (a) 1 A g^{-1}, (b) 2 A g^{-1}, (c) 5 A g^{-1} and (d) 10 A g^{-1} during galvanostatic charge and dischrage process.

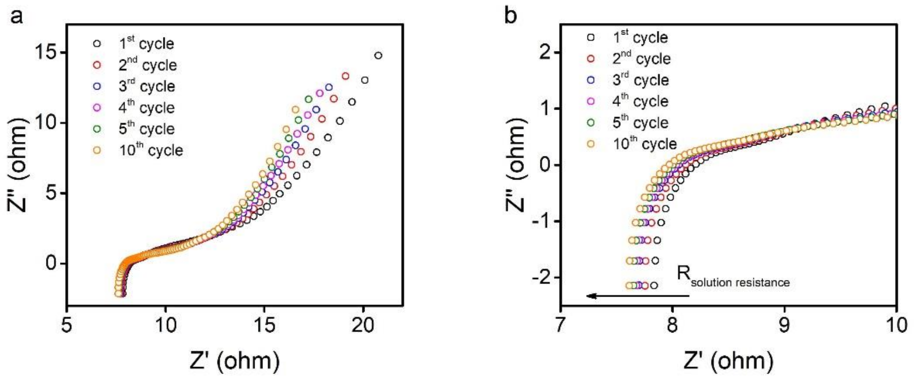

Supplementary Figure 21. EIS tests during cycling in full-cell. Electrochemical impedance measurements with (a) Nyquist plots collected at open circuit after each charge and discharge cycle of $Na_{0.27}MnO_2$ in full cell; (b) high frequency domain showing a decreased solution resistance upon cycling.

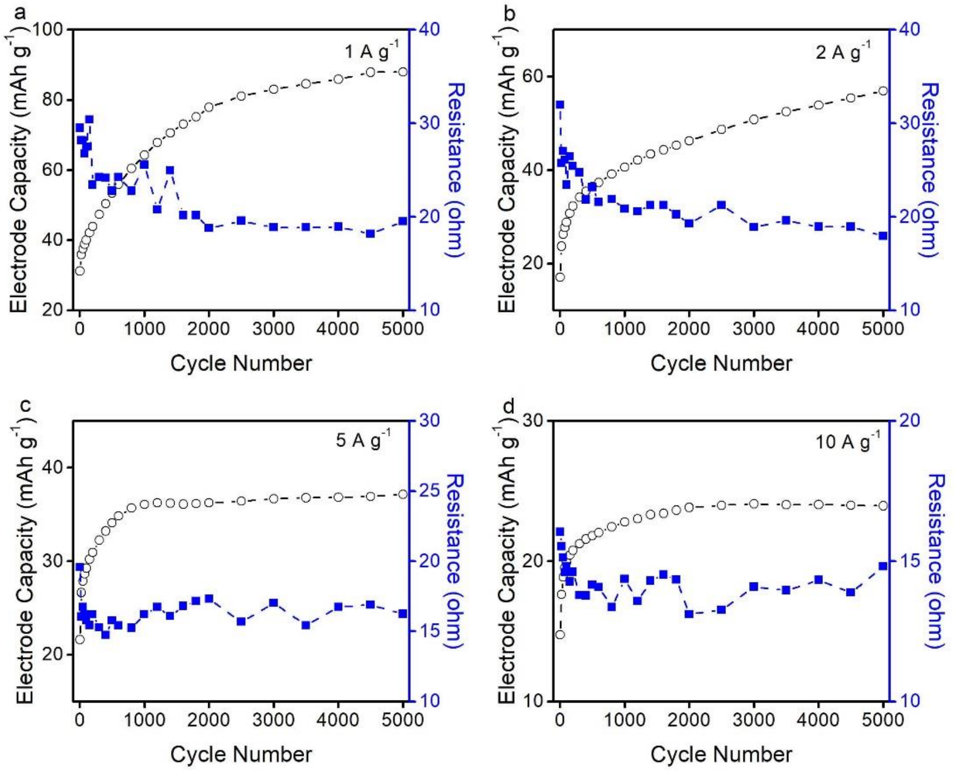

Supplementary Figure 22. Electrode capacities and cell resistances of $Na_{0.27}MnO_2$ electrode during galvanostatic charge and discharge measurements as a function of cycle numbers at the current densities of 1 A g^{-1}, 2 A g^{-1}, 5 A g^{-1} and 10 A g^{-1}.

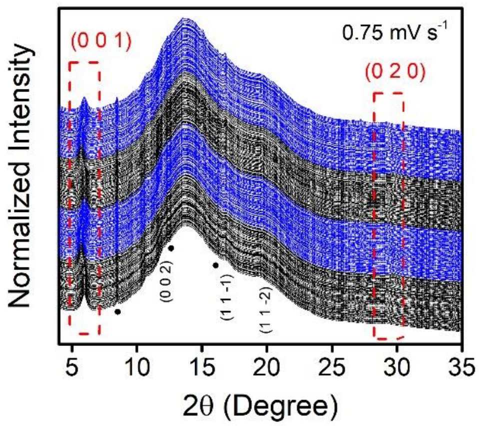

Supplementary Figure 23. *In situ* XRD patterns of $Na_{0.27}MnO_2$ obtained during 2-cycle CV measurement in $Na_{0.27}MnO_2$ electrode materials.

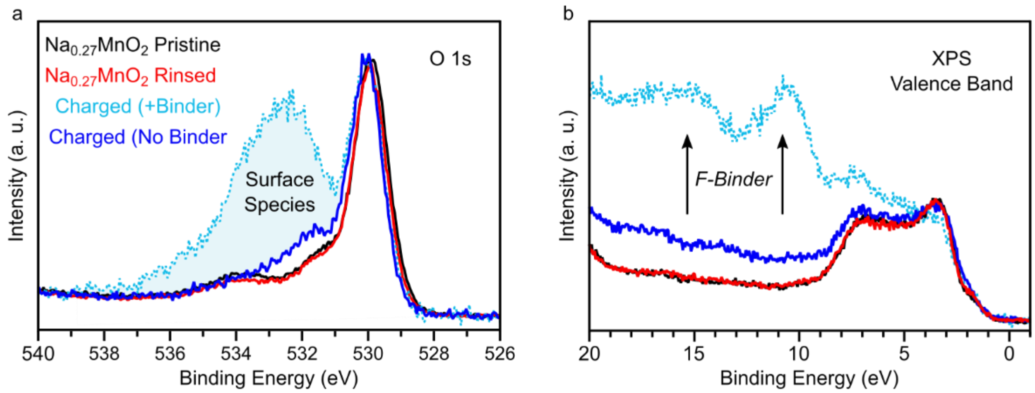

Supplementary Figure 24. XPS measurements. XPS measurements of the (a) O 1s core level and (b) valence band region for pristine and rinsed $Na_{0.27}MnO_2$ compared with $Na_{0.27}MnO_2$ electrode (with and without F-binder) charged to 1.25 V (vs Ag/AgCl). For the $Na_{0.27}MnO_2$ electrode prepared with the binder, there is an increase in the oxygen surface species and F-binder associated peaks in the valence band region.

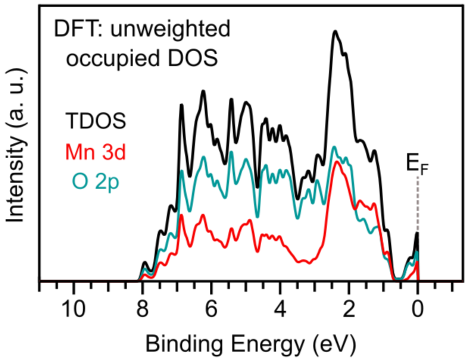

Supplementary Figure 25. Density functional theory calculations (DFT) of the occupied total density of states (TDOS) and the Mn 3d and O 2p partial density of states (PDOS). The DFT calculations predict that the highest occupied states are predominantly O 2p character which is an indication of strong covalency between the O 2p and Mn 3d states.

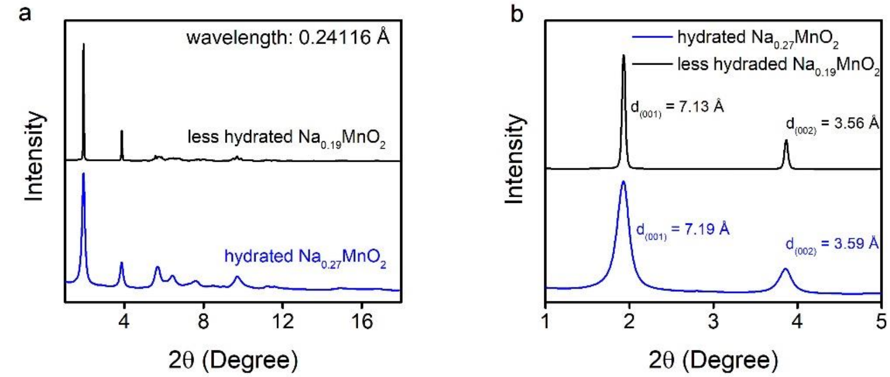

Supplementary Figure 26. XRD of hydrated and less hydrated birnessite materials. (a) XRD patterns of $Na_{0.27}MnO_2$ and less hydrated $Na_{0.19}MnO_2$ birnessite; (b) the (001) and (002) basal diffraction peaks of these two birneesite materials.

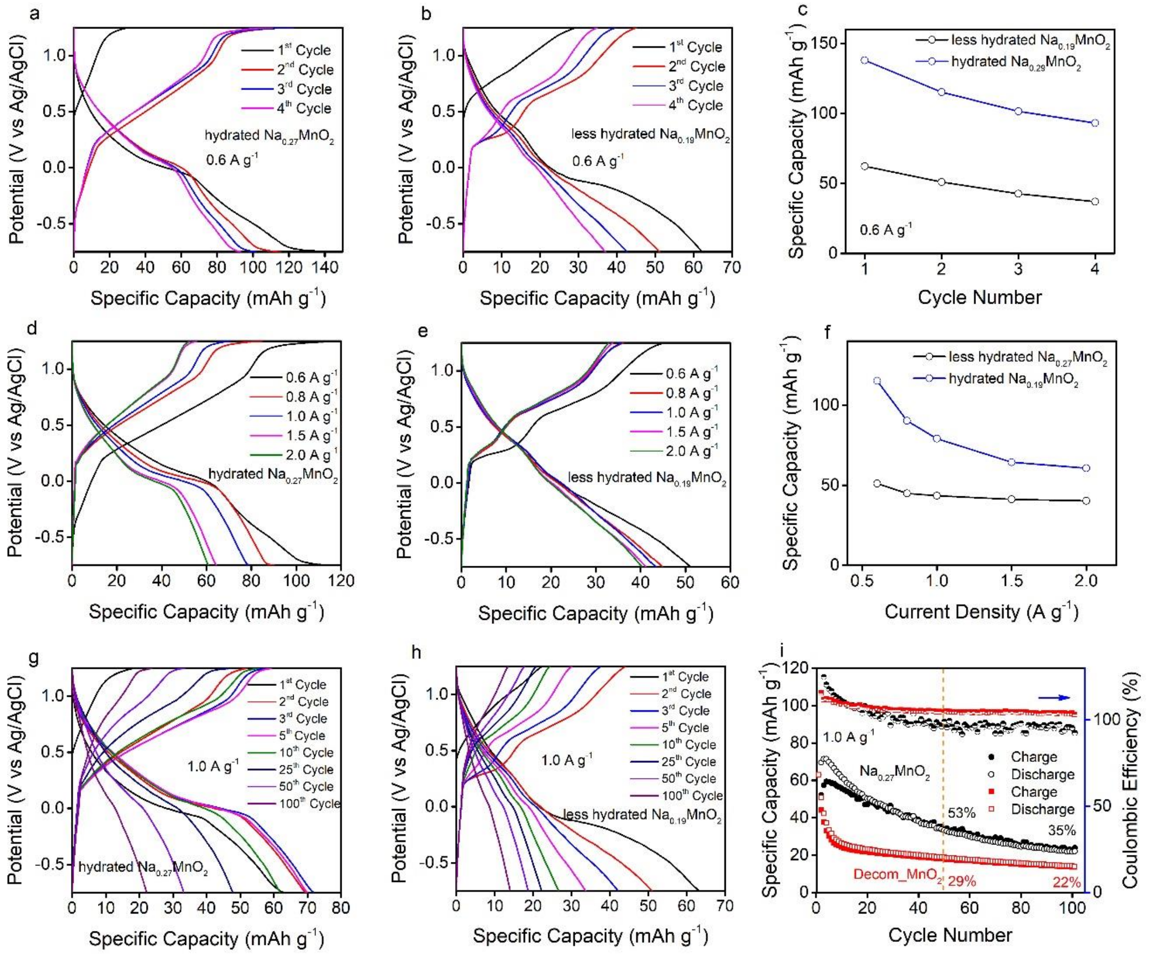

Supplementary Figure 27. CP measurements of sodium birnessite materials. (a,b,c) The initial four charge and discharge curves of hydrated $Na_{0.27}MnO_2$ and less hydrated $Na_{0.19}MnO_2$ birnessite, and the summarized specific capacities vs. cycle number; (d,e,f) the charge and discharge curves (2nd cycle) of hydrated $Na_{0.27}MnO_2$ and less hydrated $Na_{0.19}MnO_2$ birnessite at current densities from 0.6 to 2.0 A g^{-1}, and the summarized specific capacities vs. current density; (g,h,i) the charge and discharge curves of hydrated $Na_{0.27}MnO_2$ and less hydrated $Na_{0.19}MnO_2$ birnessite at 1 A g^{-1} with cycle 1st, 2nd, 3rd 5th, 10th, 25th, 50th and 100th, and the specific capacities and coulombic efficiencies vs. cycle number.

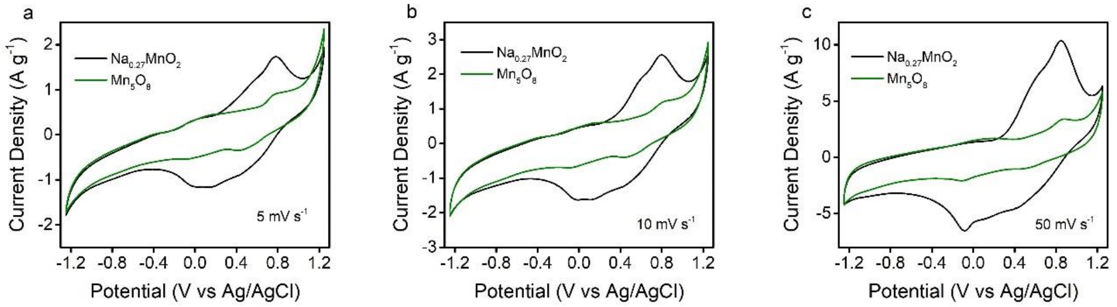

Supplementary Figure 28. CV scans of Mn_5O_8 and $Na_{0.27}MnO_2$ at the scan rates of 5, 10 and 50 mV s^{-1} between −1.25 V to 1.25 V (vs Ag/AgCl) in 0.1 M Na_2SO_4 electrolyte, showing both materials can sustain a 2.5 V potential window without significant gas evolution reactions.

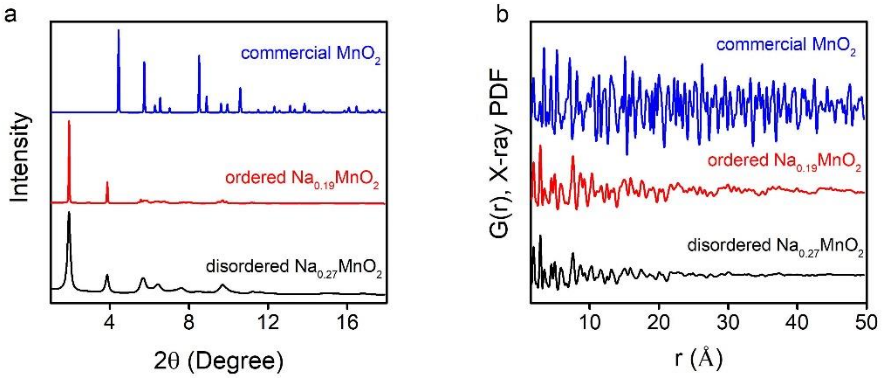

Supplementary Figure 29. XRD and X-ray PDF of disordered $Na_{0.27}MnO_2$, ordered $Na_{0.19}MnO_2$ and commercial MnO_2 materials.

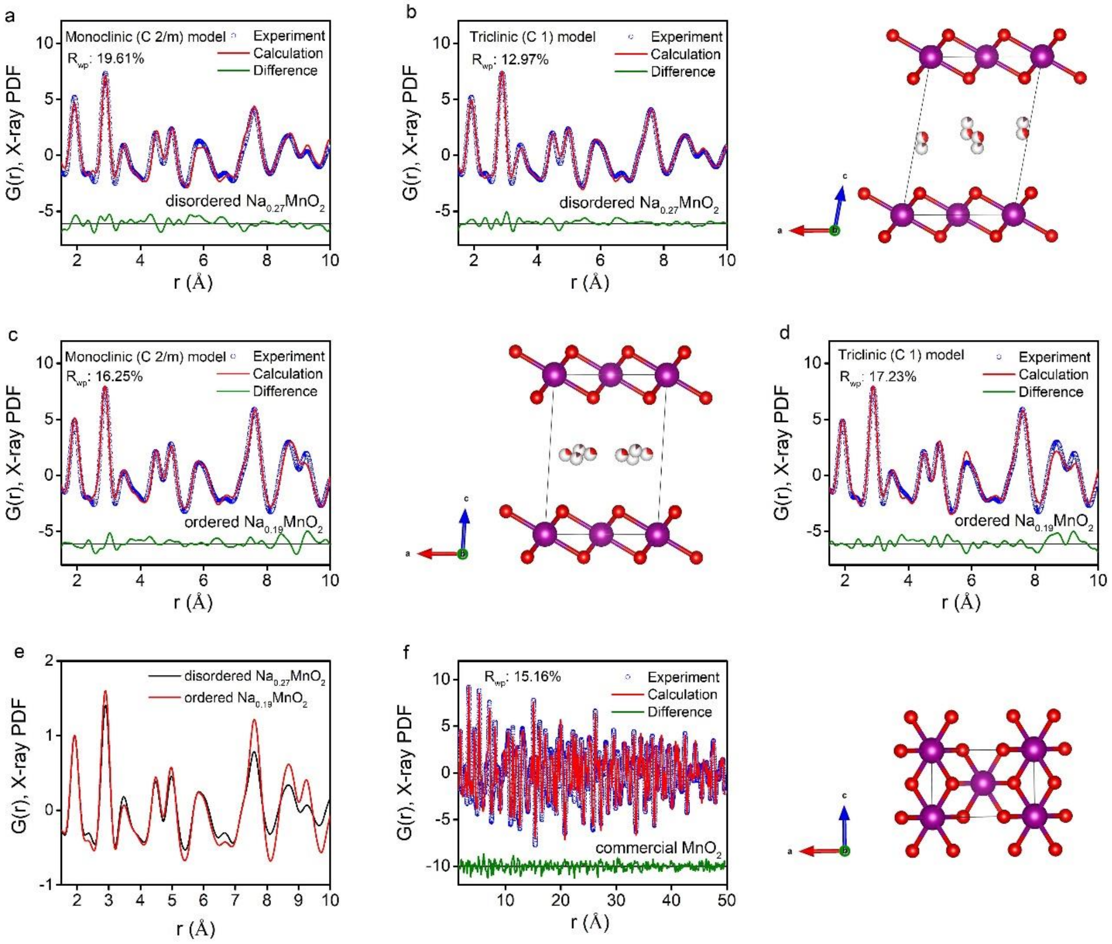

Supplementary Figure 30. XPDF analysis of MnO_2 materials. X-ray PDF fitting of disorder $Na_{0.27}MnO_2$ birnessite (a) using monoclinic (C 2/m) model with R_{wp} 19.61% and (b) using triclinic (C 1) model with R_{wp} 12.97%; X-ray PDF fitting of ordered $Na_{0.19}MnO_2$ birnessite made via thermal decomposition of $NaMnO_4$ at 800 °C (c) using monoclinc (C 2/m) model with R_{wp} 16.25% and (d) using triclinic (C 1) model with R_{wp} 17.23%; (e) the comparison of X-ray PDF of disordered $Na_{0.27}MnO_2$ and ordered $Na_{0.19}MnO_2$; and (f) X-ray PDF fitting of commercial MnO_2 with R_{wp} 15.16% with a phase of β-MnO_2.

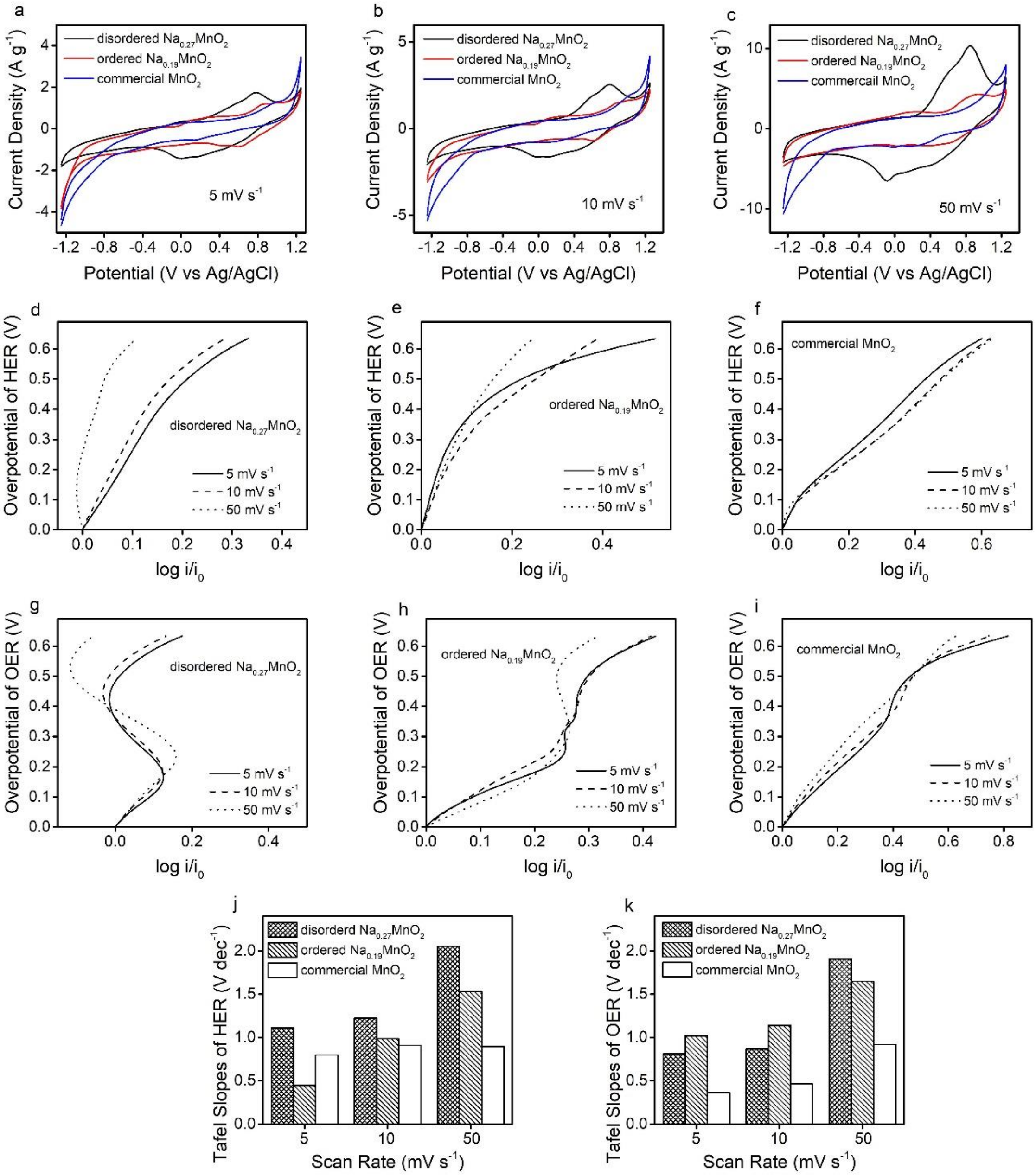

Supplementary Figure 31. HER and OER analysis of MnO_2 materials. CV scans of (a) disordered $Na_{0.27}MnO_2$, (b) ordered $Na_{0.19}MnO_2$ and (c) commercial MnO_2 materials between −1.25 V to 1.25 V (vs Ag/AgCl) in 0.1 M Na_2SO_4 electrolyte at the scan rates of 5, 10 and 50 mV s^{-1}; Tafel plots of (d, e, f) HER and (g, h, i) OER for disordered $Na_{0.27}MnO_2$, ordered $Na_{0.19}MnO_2$ and commercial MnO_2 at scan rates of 5, 10 and 50 mV s^{-1}, and the summaried Tafel slopes of (j) HER and (k) OER.

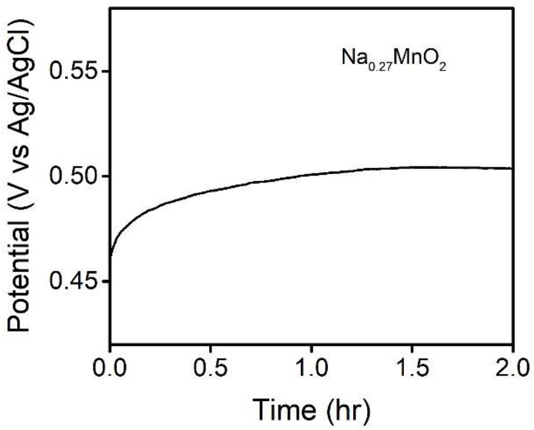

Supplementary Figure 32. The measured potential of $Na_{0.27}MnO_2$ compound at open circuit potential for 2 hours.

Supplementary Tables

Supplementary Table 1. ICP-MS results of sodium-manganese oxides ($Na_{\delta}MnO_x$) materials obtained by thermal solid-state reaction of NaOH and Mn_3O_4 with the molar ratios of 0.5:1, 1:1, 2:1, and other synthesized methods.

**Samples**	**Chemical formula determined from ICP-MS**	**Atmoic ratio of Na/Mn**
NaOH+Mn_3O_4 (0:1)	$Na_{0.01}MnO_x$	0.012±0.000
NaOH+Mn_3O_4 (0.5:1)	$Na_{0.10}MnO_x$	0.096±0.003
NaOH+Mn_3O_4 (1:1)	$Na_{0.17}MnO_x$	0.168±0.008
NaOH+Mn_3O_4 (2:1)	$Na_{0.27}MnO_2$	0.273±0.005
MnO_2 synthesized by wet chemistry method	$Na_{0.13}MnO_2$	0.134±0.003
Ordered MnO_2 synthesized by thermal decomposition of $NaMnO_4$	$Na_{0.19}MnO_2$	0.187±0.008

Supplementary Table 2. Summaried atomic ratio A/Mn (A is the cation including Na^+, K^+) of MnO_2 birnessite synthesized via a solid-state reaction compared with that of a wet chemistry method and those of other works.

Number	Sythesis Method	A/Mn	Analysis Tool	reference
**1**	Mn_3O_4 + NaOH, in air, 270 °C, 6 hours, solid-state reaction	**0.27**	ICP-MS	**Our work (Table S1)**
2	Mn^{2+} + NaOH in solution with open air	0.13	ICP-MS	**Our work (Table S1)**
3	Mn^{2+} + KOH in solution with open air	0.15	EDS	Ref. 20
4	Mn^{2+} + $K_2S_2O_8$ + NaOH in solution	0.1	ICP	Ref.21
5	Mn^{2+} + $KMnO_4$ in hydrothermal reaction, 240 °C, 3 hours	0.06	XPS (surface ratio)	Ref.22
6	$KMnO_4$ + HCl in autoclaved reaction, 140 °C, 100 min	0.12(0.01)	EDS & ICP-AES	Ref.23
7	Mn^{2+} + NaOH, air bubble	0.25	ICP-AES	Ref.24

*Note: the averaged A/Mn ratio of MnO_2 made via wet chemistry methods is about 0.14 based the work from 2 to 7.

Supplementary Table 3. Refined crystal structural parameters of Mn_5O_8 obtained by using the fitting of neutron scattering data (small-box model using PDFgui) with R_{wp} = 5.93%. The x, y, z and site indicate the atom positions and multiplicity in the unit cell, respectively. Occ. and Uiso represents the occupation and isotropic atmoic displacement parameters, respectively.

Refined crystal structural parameters of Mn_5O_8

Atom	Type	x	y	z	Occ.	Site	Uiso	U_{11}	U_{22}	U_{33}	U_{13}
Mn1	Mn+4	0.000	0.000	0.500	1.00	2c	0.01	0.014	0.014	0.014	0.005
Mn2	Mn+4	0.000	0.258	0.000	1.00	4g	0.01	0.010	0.010	0.010	0.005
Mn3	Mn+2	0.277	0.000	0.347	1.00	4i	0.02	0.020	0.020	0.020	0.005
O1	O-2	0.890	0.227	0.598	1.00	8j	0.02	0.015	0.015	0.015	0.006
O2	O-2	0.899	0.000	0.088	1.00	4i	0.02	0.015	0.015	0.015	0.006
O3	O-2	0.395	0.000	0.069	1.00	4i	0.02	0.015	0.015	0.015	0.006

Space group: C 2/m

a=10.397 b=5.725 c=4.882 (Å) α=90 β=109.816 γ=90 V=273.392 ($Å^3$)

Supplementary Table 4. Refined crystal structural parameters of $Na_{0.10}MnO_x$ (Mn_5O_8 and MnO_2) obtained by using the fitting of neutron scattering data with R_{wp} = 6.04%, showing the phase fraction of Mn_5O_8 and MnO_2 (by mass) is 56% to 44%.

Refined crystal structural parameters of Mn_5O_8

Atom	Type	x	y	z	Occ.	Site	Uiso	U_{11}	U_{22}	U_{33}	U_{13}
Mn1	Mn+4	0.000	0.000	0.500	1.00	2c	0.01	0.005	0.005	0.005	0.005
Mn2	Mn+4	0.000	0.269	0.000	1.00	4g	0.02	0.018	0.018	0.018	0.005
Mn3	Mn+2	0.262	0.000	0.349	1.00	4i	0.02	0.016	0.016	0.016	0.005
O1	O-2	0.888	0.229	0.582	1.00	8j	0.03	0.025	0.025	0.025	0.006
O2	O-2	0.901	0.000	0.095	1.00	4i	0.02	0.015	0.015	0.015	0.006
O3	O-2	0.405	0.000	0.094	1.00	4i	0.03	0.030	0.030	0.030	0.006

Space group: C 2/m

a=10.432 b=5.723 c=4.876 (Å) α=90 β=110.012 γ=90 V=273.539 ($Å^3$)

Refined crystal structural parameters of MnO_2

Atom	Type	x	y	z	Occ.	Site	Uiso	U_{11}	U_{22}	U_{33}	U_{13}
Mn1	Mn+4	0.000	0.000	0.000	1.00	2a	0.03	0.025	0.037	0.025	0.009
O1	O-2	0.386	-0.055	0.131	1.00	4i	0.05	0.025	0.043	0.090	0.037
Na1	Na+1	0.544	0.365	0.450	0.15	4i	0.01	0.009	0.002	0.006	0.002
O2	O-2	0.604	0.333	0.513	0.60	4i	0.11	0.077	0.181	0.056	0.019

Space group: C-1

a=5.058 b=2.731 c=7.387 (Å) α=87.511 β=104.993 γ=91.302 V=98.471 ($Å^3$)

Supplementary Table 5. Refined crystal structural parameters of $Na_{0.17}MnO_x$ (Mn_5O_8 and MnO_2) obtained by using the fitting of neutron scattering data with R_{wp} = 9.40%, showing the phase fraction of Mn_5O_8 and MnO_2 (by mass) is 36% to 65%.

Refined crystal structural parameters of Mn_5O_8

Atom	Type	x	y	z	Occ.	Site	Uiso	U_{11}	U_{22}	U_{33}	U_{13}
Mn1	Mn+4	0.000	0.000	0.500	1.00	2c	0.01	0.011	0.011	0.011	0.005
Mn2	Mn+4	0.000	0.281	0.000	1.00	4g	0.02	0.019	0.019	0.019	0.005
Mn3	Mn+2	0.248	0.000	0.327	1.00	4i	0.01	0.007	0.007	0.007	0.005
O1	O-2	0.888	0.230	0.595	1.00	8j	0.02	0.016	0.016	0.016	0.006
O2	O-2	0.903	0.000	0.094	1.00	4i	0.04	0.036	0.036	0.036	0.006
O3	O-2	0.428	0.000	0.080	1.00	4i	0.04	0.037	0.037	0.037	0.006

Space group: C 2/m

a=10.405 b=5.740 c=4.876 (Å) α=90 β=109.807 γ=90 V=273.954 (Å^3)

Refined crystal structural parameters of MnO_2

Atom	Type	x	y	z	Occ.	Site	Uiso	U_{11}	U_{22}	U_{33}	U_{13}
Mn1	Mn+4	0.000	0.000	0.000	1.00	2a	0.05	0.053	0.052	0.065	0.037
O1	O-2	0.391	-0.044	0.138	1.00	4i	0.05	0.025	0.036	0.089	0.036
Na1	Na+1	0.515	0.333	0.450	0.14	4i	0.01	0.007	0.002	0.011	0.003
O2	O-2	0.611	0.342	0.516	0.45	4i	0.11	0.087	0.188	0.055	0.017

Space group: C-1

a=5.070 b=2.739 c=7.363 (Å) α=86.894 β=104.782 γ=90.886 V=98.730 (Å^3)

Supplementary Table 6. Refined crystal structural parameters of $Na_{0.27}MnO_2$ (no Mn_5O_8 was observed) obtained by using the fitting of neutron scattering data with R_{wp} = 13.45%.

Refined crystal structural parameters of $Na_{0.27}MnO_2$

Atom	Type	x	y	z	Occ.	Site	Uiso	U_{11}	U_{22}	U_{33}	U_{13}
Mn1	Mn+4	0.000	0.000	0.000	1.00	2a	0.02	0.008	0.034	0.011	0.001
O1	O-2	0.384	-0.038	0.135	1.00	4i	0.05	0.027	0.046	0.089	0.036
Na1	Na+1	0.565	0.161	0.450	0.15	4i	0.01	0.009	0.002	0.006	0.002
O2	O-2	0.590	0.330	0.514	0.50	4i	0.11	0.074	0.181	0.056	0.018

Space group: C-1

a=5.048 b=2.755 c=7.381 (Å) α=86.479 β=104.175 γ=90.402 V=99.343 ($Å^3$)

Supplementary Table 7. Electrochemical peformance of our $Na_{0.27}MnO_2$ compared to other Mn based electrode materials in full cell for aqueous electrochemical energy storage.

Anode	Cathode	Electrolyte	C-rate	Voltage(V)	Electrode Capacity (mAh g^{-1})	Ref.
MnO_2/Carbon Black	MnO_2/Carbon Black	1 M Na_2SO_4	23	2.5	83	This work
$NaTi_2(PO_4)_3$	$Na_{0.44}MnO_2$	1 M Na_2SO_4	3	1.3	103	39
Mn_5O_8/Carbon Black	Mn_5O_8/Carbon Black	1M Na_2SO_4	86	3.0	106	48
Activated Carbon	MnO_2	0.1M K_2SO_4	29	2.0	51	47
$NaTi_2(PO_4)_3$	$NaMnO_2$	2M CH_3COONa	1	1.3	132	46
Graphene	MnO_2/Graphene	1M Na_2SO_4	7.4	2.0	68	43

Supplementary Table 8. Refined crystal structural parameters of disordered $Na_{0.27}MnO_2$ birnessite by X-ray scattering fitting with C-1 model with R_{wp} = 12.97%.

Refined crystal structural parameters of disordered $Na_{0.27}MnO_2$ birnessite

Atom	Type	x	y	z	Occ.	Site	Uiso	U_{11}	U_{22}	U_{33}	U_{13}
Mn1	Mn+4	0.000	0.000	0.000	1.00	2a	0.04	0.014	0.007	0.108	0.020
O1	O-2	0.623	-0.008	0.863	1.00	4i	0.02	0.023	0.006	0.430	0.020
Na1	Na+1	0.434	0.781	0.421	0.14	4i	0.06	0.006	0.021	0.137	0.015
O2	O-2	0.449	0.779	0.488	0.40	4i	0.02	0.007	0.005	0.029	0.012

Space group: C-1

a=5.097 b=2.849 c=7.362 (Å) α=87.915 β=100.481 γ=88.963 V=105.000 ($Å^3$)

Supplementary Table 9. Refined crystal structural parameters of ordered $Na_{0.19}MnO_2$ birnessite by X-ray scattering fitting with C 2/m model with R_{wp} = 16.25%.

Refined crystal structural parameters of ordered $Na_{0.19}MnO_2$ birnessite

Atom	Type	x	y	z	Occ.	Site	Uiso	U_{11}	U_{22}	U_{33}	U_{13}
Mn1	Mn+4	0.000	0.000	0.000	1.00	2a	0.07	0.021	0.010	0.176	0.074
O1	O-2	0.357	0.000	0.143	1.00	4i	0.02	0.024	0.011	0.011	0.008
Na1	Na+1	0.761	0.000	0.474	0.09	4i	0.28	0.262	0.391	0.191	0.187
O2	O-2	0.640	0.000	0.503	0.25	4i	0.08	0.009	0.211	0.006	0.001

Space group: C 2/m

a=5.029 b=2.873 c=7.140 (Å) α=90.000 β=93.178 γ=90.000 V=103.009 ($Å^3$)

Supplementary Table 10. Refined crystal structural parameters of commerical MnO_2 by X-ray scattering fitting R_{wp} = 15.16%.

Refined crystal structural parameters of commercial MnO_2

Atom	Type	x	y	z	Occ.	Site	Uiso	U_{11}	U_{22}	U_{33}	U_{13}
Mn1	Mn+4	0.000	0.000	0.000	1.00	2a	0.01	0.006	0.006	0.006	0.000
O1	O-2	0.302	0.302	0.302	1.00	4f	0.02	0.024	0.024	0.012	0.000

Space group: P 42/m n m

a=4.408 b=4.408 c=2.878 (Å) α=90 β=90 γ=90 V=55.920 ($Å^3$)

Supplementary Notes

Supplementary Note 1.

The half-cell mass specific capacitance C_{MS} (F g^{-1}) and electrode capacity $C_{electrode}$ (mAh g^{-1}) were calculated with the third CV scan.

Electrode mass specific capacitance: $C_{MS} = \frac{i}{(dV/dt)*m} = \int_{t_0}^{t_F} \frac{i}{\Delta V*m} dt$ Eq. 1

Electrode capacity: $C_{electrode} = \frac{C_{MS}*\Delta V}{3.6}$ Eq. 2

where i (A) is the measured current at certain time of t (s), m (g) is the mass of active material loaded on working electrode, ΔV (V) is potential window, t_0 (s) and t_F (s) are respective times at the initial potential and the final potential.

Supplementary Note 2.

In the manuscript, we conducted the two-electrode symmetric full-cell measurements using $Na_{0.27}MnO_2$ materials as both anode and cathode in 1M Na_2SO_4 aqueous electrolyte. During the full cell CP tests, the initial cell voltage is 0 V (namely, the potential difference between two electrodes is 0 V, and thus no redox reactions happen). When the full-cell started to discharge, one electrode (designated as M1) discharged (Na-ion insertion), while the other electrode (designated as M2) charged (Na-ion extraction) simultaneously. Reversely, when the full-cell started to charge, M1 is charged along with Na-ion extraction and M2 is discharged along with Na-ion insertion. In this context, the flux of Na-ion insertion into one electrode is always identical to the flux of Na-ion extraction from another electrode. Thus, full-cell maintained its charge neutrality as the Na-ions flowed between two electrodes, whereas the difference of the Na-ion concentration between two electrodes resulted in the difference of overall cell potential. More examples of the symmetric cell operation can also be found in recent publications. [1,2,3]

Supplementary Note 3.

The cell capacitance C_{cell} (F), cell mass-specific capacitance C_{MS} (F g^{-1}), electrode mass-specific capacitance $C_{MS(electrode)}$ (F g^{-1}), discharge cell capacity by mass $C_{discharge}$ (mAh g^{-1}), discharge electrode capacity by mass $C_{discharge(electrode)}$ (mAh g^{-1}), specific energy E_{MS} (Wh kg^{-1}), specific power P_{MS} (W kg^{-1}) and coulombic efficiency (η) and energy efficiency (γ) were calculated.

Cell capacitance: $C_{cell} = \frac{it}{U}$ Eq. 3

Cell mass-specific capacitance: $C_{MS} = \frac{it}{U*M} = \frac{it}{U*2m}$ Eq. 4

Electrode mass-specific capacitance: $C_{MS(electrode)} = 4C_{MS} = \frac{2it}{U*M/2} = \frac{2it}{U*m}$ Eq. 5

Discharge cell capacity by mass: $C_{discharge} = \frac{it}{3.6*M} = \frac{it}{3.6*2m}$ Eq. 6

Discharge electrode capacity by mass:

$C_{discharge(electrode)} = 4C_{discharge} = \frac{2it}{3.6*M/2} = \frac{2it}{3.6*m}$ Eq. 7

Specific energy: $E_{MS} = \frac{\frac{1}{2}CU^2}{3.6*M} = \frac{\frac{1}{2}*itU}{3.6*M} = \frac{\frac{1}{2}*itU}{3.6*2m}$ Eq. 8

Specific power: $P_{MS} = \frac{3600*E_{MS}}{t}$ Eq. 9

Coloumbic efficiency: $\eta = \frac{Q_{discharge}}{Q_{charge}} = \frac{it_{discharge}}{it_{charge}} = \frac{t_{discharge}}{t_{charge}}$ Eq. 10

Energy efficiency: $\gamma = \frac{\int_{V_{discharge(0)}}^{V_{discharge(F)}} C_{discharge(electrode)} dV}{\int_{V_{charge(0)}}^{V_{charge(F)}} C_{charge(electrode)} dV}$ Eq. 11

Cell resistance: $R = \frac{\Delta V}{i}$ Eq. 12

where *i* (A) is the applied constant current, *t* (s) is discharge time of the cell device, *U* (V) is potential window, ΔV (V) is the i-R drop during discharge curve, *M* (g) is the total mass of active materials on both electrodes and *m* (g) is the mass of active materials on one electrode.

Supplementary Note 4.

The current contributed from surface-controlled capacitive process can be represented by:

$i_1 = k_1 v$ Eq. 13

where *i* is the current (A) and *v* is the scan rate (mV s^{-1}).

While the current contributed from diffusion-limited redox process can be represented by:

$i_2 = k_2 v^{1/2}$ Eq. 14

Therefore, the overall current can be represented by:

$i = k_1 v + k_2 v^{1/2}$ Eq. 15

After rearrangement, it can be written as:

$\frac{i}{v^{1/2}} = k_1 v^{1/2} + k_2$ Eq. 16

By plotting $i/v^{½}$ vs. $v^{½}$ curves at a given potential, k_1 and k_2 values can be determined, and hence the current response per 0.1 V is calculated and plotted in CV and the contribution of capacitive charge and diffusion-limited redox charge during the CV measurements in half-cell can be analyzed quantitatively.

Supplementary Note 5.

The sodium diffusion kinetics can be analysed using the current-pulse relaxation technique with the following equation:

$$\frac{E_0-E}{RT} = \frac{I\Delta t}{F^2C(1-y)\sqrt{\pi D t}} \qquad \text{Eq. 17}$$

where E is the measured potential (vs Ag/AgCl), t is the time, E_0 is the open circuit potential, R is gas constant, T is absolute temperature, F is Faraday constant, I is pulse intensity, Δt is pulse duration, C is final concentration of the charge carriers, (1−y) is the occupancy rate of available sites in the structure: y = x/x_{max}.

After rearrangement, the Eq. 17 can be:

$$D = \frac{1}{k^2}\frac{1}{\pi}\left(\frac{RT*I\Delta t}{F^2C(1-y)}\right)^2 \qquad \text{Eq. 18}$$

Here $k=(E_0-E)/t^{1/2}$, and the slope k can be obtained by plotting (E_0−E) verse $t^{-1/2}$ from the relaxation period. $1-y = \Delta x/x_{max} = (I\Delta t/m)/C_{theoretical\ capacity}$, IΔt/m is a constant for all the measured $Na_\delta MnOx$ materials since the current pulse, duration and loading mass is the same, and the theoretical capacity of Mn_5O_8, $Na_{0.13}MnO_x$, $Na_{0.25}MnO_x$ and $Na_{0.29}MnO_2$ was calculated to be 200 mAh g^{-1}, 248 mAh g^{-1}, 235 mAh g^{-1} and 308 mAh g^{-1} by assuming one electron charge transfer per Mn^{4+}. Therefore, the relative ratio for Na-ion diffusion coefficient (D) of $Na_\delta MnO_x$ materials to Mn_5O_8 can be obtained since the value D is proportional to the term of $(C_{theoretical\ capacity}/k)^2$.

Supplementary Note 6.

TAFEL analyses of HER and OER were conducted in half-cell. The TAFEL equation relates the HER and OER activities to their overpotentials (η) and is expressed as:

$$\eta = A \times \log\left(\frac{i}{i_0}\right) \qquad \text{Eq. 19}$$

where *i* is experimentally observed current density, i_0 is the exchange current density (the equilibrium current density when η = 0) and A is the TAFEL slope. The TAFEL slope is calculated from the liner region of TAFEL plots (η vs. log i/i_0), typically in the overpotential range from ~ 0.5 V to ~ 0.63 V.

Supplementary Note 7.

In Supplementary Figure 8, Mn_3O_4 was converted into Mn_5O_8 through oxidation of [Mn(III)O_6] octahedra of Mn_3O_4 into [Mn(IV)O_6] units (Eq. 20), followed by Na-ion driven conversion from Mn_5O_8 to $Na_{0.27}MnO_2$ birnessite (Eq. 21) during the thermal annealing in air. It was strongly suggested that the formation of Mn_5O_8 (Eq. 20) was a much slower process compared with the formation of $Na_{0.27}MnO_2$ (Eq. 21). Therefore, when Mn_5O_8 material formed, it converted quickly into $Na_{0.27}MnO_2$ driven by the Na-ion diffusion. This hypothesis is supported by following *in situ* XRD results: (i) heating Mn_3O_4 in the air only formed Mn_5O_8 instead of $Na_{0.27}MnO_2$, indicating Na-ion is critical driving force for the formation of $Na_{0.27}MnO_2$ (Supplementary Figure 8a) (ii) a mixture of Mn_5O_8 and $Na_{0.27}MnO_2$ formed during solid-state reaction by heating Mn_3O_4 and insufficient amount NaOH and also layered MnO_2 can be formed by heating Mn_5O_8 with NaOH (Figure 1c & Supplementary 8b), indicating Mn_5O_8 is an dispensable phase

during conversion from Mn_3O_4 to $Na_{0.27}MnO_2$; (iii) during solid-state reaction between Mn_3O_4 and sufficient amount NaOH to the formation of $Na_{0.27}MnO_2$, no Mn_5O_8 phase was detected by XRD, indicating Mn_5O_8 was quickly converted into $Na_{0.27}MnO_2$ once formed (Supplementary 8c).

$$\underbrace{Mn_3O_4 \overset{O_2}{\rightarrow} Mn_5O_8}_{Slow\ Reaction} \quad \text{Eq. 20}$$

$$\underbrace{Mn_5O_8 \xrightarrow{O_2+NaOH} Na_{0.27}MnO_2}_{Fast\ Reaction} \quad \text{Eq. 21}$$

In Supplementary Figure 30, structural study including XRD and X-ray PDF analysis was conducted. X-ray PDF analysis of $Na_{0.27}MnO_2$ confirms its disordered properties with a triclinic (C-1) birnessite structure (R_{wp} =12.97%), better than a monoclinic model (R_{wp} =19.61%) with a more ordered lattice structure (Supplementary Table 8), which is also supported by our previous neutron PDF analysis. Though disordered $Na_{0.27}MnO_2$ and ordered $Na_{0.19}MnO_2$ both show the layered birnessite structure, disordered $Na_{0.27}MnO_2$ has a smaller crystalline size with shorter coherence (Supplementary Figure 29). Further X-ray PDF analysis were conducted for ordered $Na_{0.19}MnO_2$ birnessite and commercial MnO_2 as shown in Supplementary Figure 30 with strutural parameters listed in Supplementary Table 9–10, $Na_{0.19}MnO_2$ synthesized via decomposition of $NaMnO_4$ at high temperature shows a more ordered lattice structure monoclinic (C 2/m) while commercial MnO_2 is a highly crystalline β-MnO_2. The difference of local structures of disordered $Na_{0.27}MnO_2$ and ordered $Na_{0.19}MnO_2$ birnessite can be clearly observed in Supplementary Figure 30. Therefore, the smaller crystalline size with shorter coherence of $Na_{0.27}MnO_2$ as well as more disordered lattice structure is showed compared to those of $Na_{0.19}MnO_2$ birnessite and commercial MnO_2.

Supplementary References

1. Guo, S. et al. A High-Voltage and Ultralong-Life Sodium Full Cell for Stationary Energy Storage. *Angew. Chem. Int. Ed.* **40**, 11701–11705 (2015).

2. Wang, D. et al. Sodium vanadium titanium phosphate electrode for symmetric sodium-ion batteries with high power and long lifespan. *Nat. Commun.* **8**, 15888 (2017).

3. Zhang, S. & Pan, N. Supercapacitors Performance Evaluation. *Adv. Energy Mater.* **5**, 1401401 (2014).
